# Supplementary material for: Deciphering the sex bias in housekeeping gene expression in adipose tissue: a comprehensive meta-analysis of transcriptomic studies
Source: Biol Sex Differ. 2023 Apr 18;14:20. doi: 10.1186/s13293-023-00506-x (PMC10114345; doi:10.1186/s13293-023-00506-x)
Supplement: Supplementary file 1 — Additional file 1. HKG primers design process. [file 13293_2023_506_MOESM1_ESM.docx]

**Housekeeping genes (HKG) primers design process**

Genes selected as potential HKG in human and mouse WAT were *18s*, *PPIA* and *RPL19*. Primers were designed in two consecutive exons, when possible, taking into consideration all reference sequences for mRNA in NCBI (<https://www.ncbi.nlm.nih.gov/gene/>; <https://www.ncbi.nlm.nih.gov/nuccore/>) (1) and aligned to search for common regions with Pairwise Sequence Alignment **(**<https://www.ebi.ac.uk/Tools/psa/>) (2). Alternative transcript variants were analyzed by AceView (<https://www.ncbi.nlm.nih.gov/IEB/Research/Acembly/index.html>) (3) and primers (designed either by Primer3 or PrimerBlast) amplifying most represented sequence/s were chosen. Detailed sequence analysis, including reference sequences and cDNA annotation for transcript variants, for each studied HKG gene, is shown below, both for human and mouse genes.

***18S***

**Homo sapiens RNA, 18S ribosomal N5 (RNA18SN5), ribosomal RNA**

NCBI Reference Sequence: NR_003286.4

TACCTGGTTGATCCTGCCAGTAGCATATGCTTGTCTCAAAGATTAAGCCATGCATGTCTGAGTACGCACGGCCGGTACAGTGAAACTGCGAATGGCTCATTAAATCAGTTATGGTTCCTTTGGTCGCTCGCTCCTCTCCTACTTGGATAACTGTGGTAATTCTAGAGCTAATACATGCCGACGGGCGCTGACCCCCTTCGCGGGGGGGATGCGTGCATTTATCAGATCAAAACCAACCCGGTCAGCCCCTCTCCGGCCCCGGCCGGGGGGCGGGCGCCGGCGGCTTTGGTGACTCTAGATAACCTCGGGCCGATCGCACGCCCCCCGTGGCGGCGACGACCCATTCGAACGTCTGCCCTATCAACTTTCGATGGTAGTCGCCGTGCCTACCATGGTGACCACGGGTGACGGGGAATCAGGGTTCGATTCCGGAGAGGGAGCCTGAGAAACGGCTACCACATCCAAGGAAGGCAGCAGGCGCGCAAATTACCCACTCCCGACCCGGGGAGGTAGTGACGAAAAATAACAATACAGGACTCTTTCGAGGCCCTGTAATTGGAATGAGTCCACTTTAAATCCTTTAACGAGGATCCATTGGAGGGCAAGTCTGGTGCCAGCAGCCGCGGTAATTCCAGCTCCAATAGCGTATATTAAAGTTGCTGCAGTTAAAAAGCTCGTAGTTGGATCTTGGGAGCGGGCGGGCGGTCCGCCGCGAGGCGAGCCACCGCCCGTCCCCGCCCCTTGCCTCTCGGCGCCCCCTCGATGCTCTTAGCTGAGTGTCCCGCGGGGCCCGAAGCGTTTACTTTGAAAAAATTAGAGTGTTCAAAGCAGGCCCGAGCCGCCTGGATACCGCAGCTAGGAATAATGGAATAGGACCGCGGTTCTATTTTGTTGGTTTTCGGAACTGAGGCCATGATTAAGAGGGACGGCCGGGGGCATTCGTATTGCGCCGCTAGAGGTGAAATTCTTGGACCGGCGCAAGACGGACCAGAGCGAAAGCATTTGCCAAGAATGTTTTCATTAATCAAGAACGAAAGTCGGAGGTTCGAAGACGATCAGATACCGTCGTAGTTCCGACCATAAACGATGCCGACCGGCGATGCGGCGGCGTTATTCCCATGACCCGCCGGGCAGCTTCCGGGAAACCAAAGTCTTTGGGTTCCGGGGGGAGTATGGTTGCAAAGCTGAAACTTAAAGGAATTGACGGAAGGGCACCACCAGGAGTGGAGCCTGCGGCTTAATTTGACTCAACACGGGAAACCTCACCCGGCCCGGACACGGACAGGATTGACAGATTGATAGCTCTTTCTCGATTCCGTGGGTGGTGGTGCATGGCCGTTCTTAGTTGGTGGAGCGATTTGTCTGGTTAATTCCGATAACGAACGAGACTCTGGCATGCTAACTAGTTACGCGACCCCCGAGCGGTCGGCGTCCCCCAACTTCTTAGAGGGACAAGTGGCGTTCAGCCACCCGAGATTGAGCAATAACAGGTCTGTGATGCCCTTAGATGTCCGGGGCTGCACGCGCGCTACACTGACTGGCTCAGCGTGTGCCTACCCTACGCCGGCAGGCGCGGGTAACCCGTTGAACCCCATTCGTGATGGGGATCGGGGATTGCAATTATTCCCCATGAACGAGGAATTCCCAGTAAGTGCGGGTCATAAGCTTGCGTTGATTAAGTCCCTGCCCTTTGTACACACCGCCCGTCGCTACTACCGATTGGATGGTTTAGTGAGGCCCTCGGATCGGCCCCGCCGGGGTCGGCCCACGGCCCTGGCGGAGCGCTGAGAAGACGGTCGAACTTGACTATCTAGAGGAAGTAAAAGTCGTAACAAGGTTTCCGTAGGTGAACCTGCGGAAGGATCATTA

*RNA18SN5* sequence does not contain any intron, nor isoforms neither splicing variants were found. Primers selected for are highlighted in blue.

**Mus musculus 18S ribosomal RNA (Rn18s), ribosomal RNA**

NCBI Reference Sequence: NR_003278.3

ACCTGGTTGATCCTGCCAGGTAGCATATGCTTGTCTCAAAGATTAAGCCATGCATGTCTAAGTACGCACGGCCGGTACAGTGAAACTGCGAATGGCTCATTAAATCAGTTATGGTTCCTTTGGTCGCTCGCTCCTCTCCTACTTGGATAACTGTGGTAATTCTAGAGCTAATACATGCCGACGGGCGCTGACCCCCCTTCCCGGGGGGGGATGCGTGCATTTATCAGATCAAAACCAACCCGGTGAGCTCCCTCCCGGCTCCGGCCGGGGGTCGGGCGCCGGCGGCTTGGTGACTCTAGATAACCTCGGGCCGATCGCACGCCCCCCGTGGCGGCGACGACCCATTCGAACGTCTGCCCTATCAACTTTCGATGGTAGTCGCCGTGCCTACCATGGTGACCACGGGTGACGGGGAATCAGGGTTCGATTCCGGAGAGGGAGCCTGAGAAACGGCTACCACATCCAAGGAAGGCAGCAGGCGCGCAAATTACCCACTCCCGACCCGGGGAGGTAGTGACGAAAAATAACAATACAGGACTCTTTCGGGCCCTGTAATTGGAATGAGTCCACTTTAAATCCTTTAACGAGGATCCATTGGAGGGCAAGTCTGGTGCCAGCAGCCGCGGTAATTCCAGCTCCAATAGCGTATATTAAAGTTGCTGCAGTTAAAAAGCTCGTAGTTGGATCTTGGGAGCGGGCGGGCGGTCCGCCGCGAGGCGAGTCACCGCCCGTCCCCGCCCCTTGCCTCTCGGCGCCCCCTCGATGCTCTTAGCTGAGTGTCCCGCGGGGCCCGAAGCGTTTACTTTGAAAAAATTAGAGTGTTCAAAGCAGGCCCGAGCCGCCTGGATACCGCAGCTAGGAATAATGGAATAGGACCGCGGTTCTATTTTGTTGGTTTTCGGAACTGAGGCCATGATTAAGAGGGACGGCCGGGGGCATTCGTATTGCGCCGCTAGAGGTGAAATTCTTGGACCGGCGCAAGACGGACCAGAGCGAAAGCATTTGCCAAGAATGTTTTCATTAATCAAGAACGAAAGTCGGAGGTTCGAAGACGATCAGATACCGTCGTAGTTCCGACCATAAACGATGCCGACTGGCGATGCGGCGGCGTTATTCCCATGACCCGCCGGGCAGCTTCCGGGAAACCAAAGTCTTTGGGTTCCGGGGGGAGTATGGTTGCAAAGCTGAAACTTAAAGGAATTGACGGAAGGGCACCACCAGGAGTGGGCCTGCGGCTTAATTTGACTCAACACGGGAAACCTCACCCGGCCCGGACACGGACAGGATTGACAGATTGATAGCTCTTTCTCGATTCCGTGGGTGGTGGTGCATGGCCGTTCTTAGTTGGTGGAGCGATTTGTCTGGTTAATTCCGATAACGAACGAGACTCTGGCATGCTAACTAGTTACGCGACCCCCGAGCGGTCGGCGTCCCCCAACTTCTTAGAGGGACAAGTGGCGTTCAGCCACCCGAGATTGAGCAATAACAGGTCTGTGATGCCCTTAGATGTCCGGGGCTGCACGCGCGCTACACTGACTGGCTCAGCGTGTGCCTACCCTGCGCCGGCAGGCGCGGGTAACCCGTTGAACCCCATTCGTGATGGGGATCGGGGATTGCAATTATTCCCCATGAACGAGGAATTCCCAGTAAGTGCGGGTCATAAGCTTGCGTTGATTAAGTCCCTGCCCTTTGTACACACCGCCCGTCGCTACTACCGATTGGATGGTTTAGTGAGGCCCTCGGATCGGCCCCGCCGGGGTCGGCCCACGGCCCTGGCGGAGCGCTGAGAAGACGGTCGAACTTGACTATCTAGAGGAAGTAAAAGTCGTAACAAGGTTTCCGTAGGTGAACCTGCGGAAGGATCATTAA

*RN18s* sequence does not contain any intron, nor isoforms neither splicing variants were found. Primers selected for are highlighted in blue.

***PPIA***

**PPIA mRNA SEQUENCE (Homo sapiens)**

According to NCBI gene page:

<https://www.ncbi.nlm.nih.gov/gene/5478>

PPIA has 2 reference Sequences for mRNA and Protein(s):

[**NM_001300981.2**](https://www.ncbi.nlm.nih.gov/nuccore/NM_001300981.2)**→**[**NP_001287910.1**](https://www.ncbi.nlm.nih.gov/protein/NP_001287910.1)**peptidyl-prolyl cis-trans isomerase A isoform 2**

[**NM_021130.5**](https://www.ncbi.nlm.nih.gov/nuccore/NM_021130.5)**→**[**NP_066953.1**](https://www.ncbi.nlm.nih.gov/protein/NP_066953.1)**peptidyl-prolyl cis-trans isomerase A isoform 1**

**Using EMBOSS Needle Tool:**

<https://www.ebi.ac.uk/Tools/services/web/toolresult.ebi?jobId=emboss_needle-I20221005-123108-0903-55648832-p2m>

**Pairwise sequence alignement has been performed and common sequence for both isoforms is a s follows:**

########################################

# Program: needle

# Rundate: Wed 5 Oct 2022 12:31:10

# Commandline: needle

# -auto

# -stdout

# -asequence emboss_needle-I20221005-123108-0903-55648832-p2m.asequence

# -bsequence emboss_needle-I20221005-123108-0903-55648832-p2m.bsequence

# -datafile EDNAFULL

# -gapopen 10.0

# -gapextend 0.5

# -endopen 10.0

# -endextend 0.5

# -aformat3 pair

# -snucleotide1

# -snucleotide2

# Align_format: pair

# Report_file: stdout

########################################

#=======================================

#

# Aligned_sequences: 2

# 1: NM_001300981.2

# 2: NM_021130.5

# Matrix: EDNAFULL

# Gap_penalty: 10.0

# Extend_penalty: 0.5

#

# Length: 2394

# Identity: 2237/2394 (93.4%)

# Similarity: 2237/2394 (93.4%)

# Gaps: 157/2394 ( 6.6%)

# Score: 11097.0

#

#

#=======================================

NM_001300981. 1 GTTTTGCAGACGCCACCGCCGAGGAAAACCGTGTACTATTAGCCATGGTC 50

||||||||||||||||||||||||||||||||||||||||||||||||||

NM_021130.5 1 GTTTTGCAGACGCCACCGCCGAGGAAAACCGTGTACTATTAGCCATGGTC 50

NM_001300981. 51 AACCCCACCGTGTTCTTCGACATTGCCGTCGACGGCGAGCCCTTGGGCCG 100

||||||||||||||||||||||||||||||||||||||||||||||||||

NM_021130.5 51 AACCCCACCGTGTTCTTCGACATTGCCGTCGACGGCGAGCCCTTGGGCCG 100

NM_001300981. 101 CGTCTCCTTTGAGGTAAGGGGCCTGGATACCAAGAAGTGACTGCTCATCT 150

|||||||||||||

NM_021130.5 101 CGTCTCCTTTGAG------------------------------------- 113

NM_001300981. 151 AATCCATAAAGCTATGTTAACAGATTGGAGGTAGTAGCATTTTCATTACA 200

NM_021130.5 114 -------------------------------------------------- 113

NM_001300981. 201 AGTGACTAAAAGAACAGCTGTTTACCCCTGATCGTGCAGCAGTGCTTGCT 250

NM_021130.5 114 -------------------------------------------------- 113

NM_001300981. 251 GTTCCTTAGAATTTTGCCTTCTGTTTGCAGACAAGGTCCCAAAGACAGCA 300

||||||||||||||||||||||||||||||

NM_021130.5 114 --------------------CTGTTTGCAGACAAGGTCCCAAAGACAGCA 143

NM_001300981. 301 GAAAATTTTCGTGCTCTGAGCACTGGAGAGAAAGGATTTGGTTATAAGGG 350

||||||||||||||||||||||||||||||||||||||||||||||||||

NM_021130.5 144 GAAAATTTTCGTGCTCTGAGCACTGGAGAGAAAGGATTTGGTTATAAGGG 193

NM_001300981. 351 TTCCTGCTTTCACAGAATTATTCCAGGGTTTATGTGTCAGGGTGGTGACT 400

||||||||||||||||||||||||||||||||||||||||||||||||||

NM_021130.5 194 TTCCTGCTTTCACAGAATTATTCCAGGGTTTATGTGTCAGGGTGGTGACT 243

NM_001300981. 401 TCACACGCCATAATGGCACTGGTGGCAAGTCCATCTATGGGGAGAAATTT 450

||||||||||||||||||||||||||||||||||||||||||||||||||

NM_021130.5 244 TCACACGCCATAATGGCACTGGTGGCAAGTCCATCTATGGGGAGAAATTT 293

NM_001300981. 451 GAAGATGAGAACTTCATCCTAAAGCATACGGGTCCTGGCATCTTGTCCAT 500

||||||||||||||||||||||||||||||||||||||||||||||||||

NM_021130.5 294 GAAGATGAGAACTTCATCCTAAAGCATACGGGTCCTGGCATCTTGTCCAT 343

NM_001300981. 501 GGCAAATGCTGGACCCAACACAAATGGTTCCCAGTTTTTCATCTGCACTG 550

||||||||||||||||||||||||||||||||||||||||||||||||||

NM_021130.5 344 GGCAAATGCTGGACCCAACACAAATGGTTCCCAGTTTTTCATCTGCACTG 393

NM_001300981. 551 CCAAGACTGAGTGGTTGGATGGCAAGCATGTGGTGTTTGGCAAAGTGAAA 600

||||||||||||||||||||||||||||||||||||||||||||||||||

NM_021130.5 394 CCAAGACTGAGTGGTTGGATGGCAAGCATGTGGTGTTTGGCAAAGTGAAA 443

NM_001300981. 601 GAAGGCATGAATATTGTGGAGGCCATGGAGCGCTTTGGGTCCAGGAATGG 650

||||||||||||||||||||||||||||||||||||||||||||||||||

NM_021130.5 444 GAAGGCATGAATATTGTGGAGGCCATGGAGCGCTTTGGGTCCAGGAATGG 493

NM_001300981. 651 CAAGACCAGCAAGAAGATCACCATTGCTGACTGTGGACAACTCGAATAAG 700

||||||||||||||||||||||||||||||||||||||||||||||||||

NM_021130.5 494 CAAGACCAGCAAGAAGATCACCATTGCTGACTGTGGACAACTCGAATAAG 543

NM_001300981. 701 TTTGACTTGTGTTTTATCTTAACCACCAGATCATTCCTTCTGTAGCTCAG 750

||||||||||||||||||||||||||||||||||||||||||||||||||

NM_021130.5 544 TTTGACTTGTGTTTTATCTTAACCACCAGATCATTCCTTCTGTAGCTCAG 593

NM_001300981. 751 GAGAGCACCCCTCCACCCCATTTGCTCGCAGTATCCTAGAATCTTTGTGC 800

||||||||||||||||||||||||||||||||||||||||||||||||||

NM_021130.5 594 GAGAGCACCCCTCCACCCCATTTGCTCGCAGTATCCTAGAATCTTTGTGC 643

NM_001300981. 801 TCTCGCTGCAGTTCCCTTTGGGTTCCATGTTTTCCTTGTTCCCTCCCATG 850

||||||||||||||||||||||||||||||||||||||||||||||||||

NM_021130.5 644 TCTCGCTGCAGTTCCCTTTGGGTTCCATGTTTTCCTTGTTCCCTCCCATG 693

NM_001300981. 851 CCTAGCTGGATTGCAGAGTTAAGTTTATGATTATGAAATAAAAACTAAAT 900

||||||||||||||||||||||||||||||||||||||||||||||||||

NM_021130.5 694 CCTAGCTGGATTGCAGAGTTAAGTTTATGATTATGAAATAAAAACTAAAT 743

NM_001300981. 901 AACAATTGTCCTCGTTTGAGTTAAGAGTGTTGATGTAGGCTTTATTTTAA 950

||||||||||||||||||||||||||||||||||||||||||||||||||

NM_021130.5 744 AACAATTGTCCTCGTTTGAGTTAAGAGTGTTGATGTAGGCTTTATTTTAA 793

NM_001300981. 951 GCAGTAATGGGTTACTTCTGAAACATCACTTGTTTGCTTAATTCTACACA 1000

||||||||||||||||||||||||||||||||||||||||||||||||||

NM_021130.5 794 GCAGTAATGGGTTACTTCTGAAACATCACTTGTTTGCTTAATTCTACACA 843

NM_001300981. 1001 GTACTTAGATTTTTTTTACTTTCCAGTCCCAGGAAGTGTCAATGTTTGTT 1050

||||||||||||||||||||||||||||||||||||||||||||||||||

NM_021130.5 844 GTACTTAGATTTTTTTTACTTTCCAGTCCCAGGAAGTGTCAATGTTTGTT 893

NM_001300981. 1051 GAGTGGAATATTGAAAATGTAGGCAGCAACTGGGCATGGTGGCTCACTGT 1100

||||||||||||||||||||||||||||||||||||||||||||||||||

NM_021130.5 894 GAGTGGAATATTGAAAATGTAGGCAGCAACTGGGCATGGTGGCTCACTGT 943

NM_001300981. 1101 CTGTAATGTATTACCTGAGGCAGAAGACCACCTGAGGGTAGGAGTCAAGA 1150

||||||||||||||||||||||||||||||||||||||||||||||||||

NM_021130.5 944 CTGTAATGTATTACCTGAGGCAGAAGACCACCTGAGGGTAGGAGTCAAGA 993

NM_001300981. 1151 TCAGCCTGGGCAACATAGTGAGACGCTGTCTCTACAAAAAATAATTAGCC 1200

||||||||||||||||||||||||||||||||||||||||||||||||||

NM_021130.5 994 TCAGCCTGGGCAACATAGTGAGACGCTGTCTCTACAAAAAATAATTAGCC 1043

NM_001300981. 1201 TGGCCTGGTGGTGCATGCCTAGTCCTAGCTGATCTGGAGGCTGACGTGGG 1250

||||||||||||||||||||||||||||||||||||||||||||||||||

NM_021130.5 1044 TGGCCTGGTGGTGCATGCCTAGTCCTAGCTGATCTGGAGGCTGACGTGGG 1093

NM_001300981. 1251 AGGATTGCTTGAGCCTAGAGTGAGCTATTATCATGCCACTGTACAGCCTG 1300

||||||||||||||||||||||||||||||||||||||||||||||||||

NM_021130.5 1094 AGGATTGCTTGAGCCTAGAGTGAGCTATTATCATGCCACTGTACAGCCTG 1143

NM_001300981. 1301 GGTGTTCACAGATCTTGTGTCTCAAAGGTAGGCAGAGGCAGGAAAAGCAA 1350

||||||||||||||||||||||||||||||||||||||||||||||||||

NM_021130.5 1144 GGTGTTCACAGATCTTGTGTCTCAAAGGTAGGCAGAGGCAGGAAAAGCAA 1193

NM_001300981. 1351 GGAGCCAGAATTAAGAGGTTGGGTCAGTCTGCAGTGAGTTCATGCATTTA 1400

||||||||||||||||||||||||||||||||||||||||||||||||||

NM_021130.5 1194 GGAGCCAGAATTAAGAGGTTGGGTCAGTCTGCAGTGAGTTCATGCATTTA 1243

NM_001300981. 1401 GAGGTGTTCTTCAAGATGACTAATGTCAAAAATTGAGACATCTGTTGCGG 1450

||||||||||||||||||||||||||||||||||||||||||||||||||

NM_021130.5 1244 GAGGTGTTCTTCAAGATGACTAATGTCAAAAATTGAGACATCTGTTGCGG 1293

NM_001300981. 1451 TTTTTTTTTTTTTTTTTTCCCCTGGAATGCAGTGGCGTGATCTCAGCTCA 1500

||||||||||||||||||||||||||||||||||||||||||||||||||

NM_021130.5 1294 TTTTTTTTTTTTTTTTTTCCCCTGGAATGCAGTGGCGTGATCTCAGCTCA 1343

NM_001300981. 1501 CTGCAGCCTCCGCCTCCTGGGTTCAAGTGATTCTAGTGCCTCAGCCTCCT 1550

||||||||||||||||||||||||||||||||||||||||||||||||||

NM_021130.5 1344 CTGCAGCCTCCGCCTCCTGGGTTCAAGTGATTCTAGTGCCTCAGCCTCCT 1393

NM_001300981. 1551 GAGTAGCTGGGATAATGGGCGTGTGCCACCATGCCCAGCTAATTTTTGTA 1600

||||||||||||||||||||||||||||||||||||||||||||||||||

NM_021130.5 1394 GAGTAGCTGGGATAATGGGCGTGTGCCACCATGCCCAGCTAATTTTTGTA 1443

NM_001300981. 1601 TTTTTAGTATAGATGGGGTTTCATCATTTTGACCAGGCTGGTCTCAAACT 1650

||||||||||||||||||||||||||||||||||||||||||||||||||

NM_021130.5 1444 TTTTTAGTATAGATGGGGTTTCATCATTTTGACCAGGCTGGTCTCAAACT 1493

NM_001300981. 1651 CTTGACCTCAGCTGATGCGCCTGCCTTGGCCTCCCAAACTGCTGAGATTA 1700

||||||||||||||||||||||||||||||||||||||||||||||||||

NM_021130.5 1494 CTTGACCTCAGCTGATGCGCCTGCCTTGGCCTCCCAAACTGCTGAGATTA 1543

NM_001300981. 1701 CAGATGTGAGCCACCGCACCCTACCTCATTTTCTGTAACAAAGCTAAGCT 1750

||||||||||||||||||||||||||||||||||||||||||||||||||

NM_021130.5 1544 CAGATGTGAGCCACCGCACCCTACCTCATTTTCTGTAACAAAGCTAAGCT 1593

NM_001300981. 1751 TGAACACTGTTGATGTTCTTGAGGGAAGCATATTGGGCTTTAGGCTGTAG 1800

||||||||||||||||||||||||||||||||||||||||||||||||||

NM_021130.5 1594 TGAACACTGTTGATGTTCTTGAGGGAAGCATATTGGGCTTTAGGCTGTAG 1643

NM_001300981. 1801 GTCAAGTTTATACATCTTAATTATGGTGGAATTCCTATGTAGAGTCTAAA 1850

||||||||||||||||||||||||||||||||||||||||||||||||||

NM_021130.5 1644 GTCAAGTTTATACATCTTAATTATGGTGGAATTCCTATGTAGAGTCTAAA 1693

NM_001300981. 1851 AAGCCAGGTACTTGGTGCTACAGTCAGTCTCCCTGCAGAGGGTTAAGGCG 1900

||||||||||||||||||||||||||||||||||||||||||||||||||

NM_021130.5 1694 AAGCCAGGTACTTGGTGCTACAGTCAGTCTCCCTGCAGAGGGTTAAGGCG 1743

NM_001300981. 1901 CAGACTACCTGCAGTGAGGAGGTACTGCTTGTAGCATATAGAGCCTCTCC 1950

||||||||||||||||||||||||||||||||||||||||||||||||||

NM_021130.5 1744 CAGACTACCTGCAGTGAGGAGGTACTGCTTGTAGCATATAGAGCCTCTCC 1793

NM_001300981. 1951 CTAGCTTTGGTTATGGAGGCTTTGAGGTTTTGCAAACCTGACCAATTTAA 2000

||||||||||||||||||||||||||||||||||||||||||||||||||

NM_021130.5 1794 CTAGCTTTGGTTATGGAGGCTTTGAGGTTTTGCAAACCTGACCAATTTAA 1843

NM_001300981. 2001 GCCATAAGATCTGGTCAAAGGGATACCCTTCCCACTAAGGACTTGGTTTC 2050

||||||||||||||||||||||||||||||||||||||||||||||||||

NM_021130.5 1844 GCCATAAGATCTGGTCAAAGGGATACCCTTCCCACTAAGGACTTGGTTTC 1893

NM_001300981. 2051 TCAGGAAATTATATGTACAGTGCTTGCTGGCAGTTAGATGTCAGGACAAT 2100

||||||||||||||||||||||||||||||||||||||||||||||||||

NM_021130.5 1894 TCAGGAAATTATATGTACAGTGCTTGCTGGCAGTTAGATGTCAGGACAAT 1943

NM_001300981. 2101 CTAAGCTGAGAAAACCCCTTCTCTGCCCACCTTAACAGACCTCTAGGGTT 2150

||||||||||||||||||||||||||||||||||||||||||||||||||

NM_021130.5 1944 CTAAGCTGAGAAAACCCCTTCTCTGCCCACCTTAACAGACCTCTAGGGTT 1993

NM_001300981. 2151 CTTAACCCAGCAATCAAGTTTGCCTATCCTAGAGGTGGCGGATTTGATCA 2200

||||||||||||||||||||||||||||||||||||||||||||||||||

NM_021130.5 1994 CTTAACCCAGCAATCAAGTTTGCCTATCCTAGAGGTGGCGGATTTGATCA 2043

NM_001300981. 2201 TTTGGTGTGTTGGGCAATTTTTGTTTTACTGTCTGGTTCCTTCTGCGTGA 2250

||||||||||||||||||||||||||||||||||||||||||||||||||

NM_021130.5 2044 TTTGGTGTGTTGGGCAATTTTTGTTTTACTGTCTGGTTCCTTCTGCGTGA 2093

NM_001300981. 2251 ATTACCACCACCACCACTTGTGCATCTCAGTCTTGTGTGTTGTCTGGTTA 2300

||||||||||||||||||||||||||||||||||||||||||||||||||

NM_021130.5 2094 ATTACCACCACCACCACTTGTGCATCTCAGTCTTGTGTGTTGTCTGGTTA 2143

NM_001300981. 2301 CGTATTCCCTGGGTGATACCATTCAATGTCTTAATGTACTTGTGGCTCAG 2350

||||||||||||||||||||||||||||||||||||||||||||||||||

NM_021130.5 2144 CGTATTCCCTGGGTGATACCATTCAATGTCTTAATGTACTTGTGGCTCAG 2193

NM_001300981. 2351 ACCTGAGTGCAAGGTGGAAATAAACATCAAACATCTTTTCATTA 2394

||||||||||||||||||||||||||||||||||||||||||||

NM_021130.5 2194 ACCTGAGTGCAAGGTGGAAATAAACATCAAACATCTTTTCATTA 2237

# Homo sapiens peptidylprolyl isomerase A (PPIA), transcript variant 2, mRNA

NCBI Reference Sequence: NM_001300981.2

GTTTTGCAGACGCCACCGCCGAGGAAAACCGTGTACTATTAGCCATGGTCAACCCCACCGTGTTCTTCGACATTGCCGTCGACGGCGAGCCCTTGGGCCGCGTCTCCTTTGAGGTAAGGGGCCTGGATACCAAGAAGTGACTGCTCATCTAATCCATAAAGCTATGTTAACAGATTGGAGGTAGTAGCATTTTCATTACAAGTGACTAAAAGAACAGCTGTTTACCCCTGATCGTGCAGCAGTGCTTGCTGTTCCTTAGAATTTTGCCTTCTGTTTGCAGACAAGGTCCCAAAGACAGCAGAAAATTTTCGTGCTCTGAGCACTGGAGAGAAAGGATTTGGTTATAAGGGTTCCTGCTTTCACAGAATTATTCCAGGGTTT**ATGTGTCAGGGTGGTGACTTCACACGCCATAATGGCACTGGTGGCAAGTCCATCTATGGGGAGAAATTTGAAGATGAGAACTTCATCCTAAAGCATACGGGTCCTGGCATCTTGTCCATGGCAAATGCTGGACCCAACACAAATGGTTCCCAGTTTTTCATCTGCACTGCCAAGACTGAGTGGTTGGATGGCAAGCATGTGGTGTTTGGCAAAGTGAAAGAAGGCATGAATATTGTGGAGGCCATGGAGCGCTTTGGGTCCAGGAATGGCAAGACCAGCAAGAAGATCACCATTGCTGACTGTGGACAACTCGAATAA**GTTTGACTTGTGTTTTATCTTAACCACCAGATCATTCCTTCTGTAGCTCAGGAGAGCACCCCTCCACCCCATTTGCTCGCAGTATCCTAGAATCTTTGTGCTCTCGCTGCAGTTCCCTTTGGGTTCCATGTTTTCCTTGTTCCCTCCCATGCCTAGCTGGATTGCAGAGTTAAGTTTATGATTATGAAATAAAAACTAAATAACAATTGTCCTCGTTTGAGTTAAGAGTGTTGATGTAGGCTTTATTTTAAGCAGTAATGGGTTACTTCTGAAACATCACTTGTTTGCTTAATTCTACACAGTACTTAGATTTTTTTTACTTTCCAGTCCCAGGAAGTGTCAATGTTTGTTGAGTGGAATATTGAAAATGTAGGCAGCAACTGGGCATGGTGGCTCACTGTCTGTAATGTATTACCTGAGGCAGAAGACCACCTGAGGGTAGGAGTCAAGATCAGCCTGGGCAACATAGTGAGACGCTGTCTCTACAAAAAATAATTAGCCTGGCCTGGTGGTGCATGCCTAGTCCTAGCTGATCTGGAGGCTGACGTGGGAGGATTGCTTGAGCCTAGAGTGAGCTATTATCATGCCACTGTACAGCCTGGGTGTTCACAGATCTTGTGTCTCAAAGGTAGGCAGAGGCAGGAAAAGCAAGGAGCCAGAATTAAGAGGTTGGGTCAGTCTGCAGTGAGTTCATGCATTTAGAGGTGTTCTTCAAGATGACTAATGTCAAAAATTGAGACATCTGTTGCGGTTTTTTTTTTTTTTTTTTCCCCTGGAATGCAGTGGCGTGATCTCAGCTCACTGCAGCCTCCGCCTCCTGGGTTCAAGTGATTCTAGTGCCTCAGCCTCCTGAGTAGCTGGGATAATGGGCGTGTGCCACCATGCCCAGCTAATTTTTGTATTTTTAGTATAGATGGGGTTTCATCATTTTGACCAGGCTGGTCTCAAACTCTTGACCTCAGCTGATGCGCCTGCCTTGGCCTCCCAAACTGCTGAGATTACAGATGTGAGCCACCGCACCCTACCTCATTTTCTGTAACAAAGCTAAGCTTGAACACTGTTGATGTTCTTGAGGGAAGCATATTGGGCTTTAGGCTGTAGGTCAAGTTTATACATCTTAATTATGGTGGAATTCCTATGTAGAGTCTAAAAAGCCAGGTACTTGGTGCTACAGTCAGTCTCCCTGCAGAGGGTTAAGGCGCAGACTACCTGCAGTGAGGAGGTACTGCTTGTAGCATATAGAGCCTCTCCCTAGCTTTGGTTATGGAGGCTTTGAGGTTTTGCAAACCTGACCAATTTAAGCCATAAGATCTGGTCAAAGGGATACCCTTCCCACTAAGGACTTGGTTTCTCAGGAAATTATATGTACAGTGCTTGCTGGCAGTTAGATGTCAGGACAATCTAAGCTGAGAAAACCCCTTCTCTGCCCACCTTAACAGACCTCTAGGGTTCTTAACCCAGCAATCAAGTTTGCCTATCCTAGAGGTGGCGGATTTGATCATTTGGTGTGTTGGGCAATTTTTGTTTTACTGTCTGGTTCCTTCTGCGTGAATTACCACCACCACCACTTGTGCATCTCAGTCTTGTGTGTTGTCTGGTTACGTATTCCCTGGGTGATACCATTCAATGTCTTAATGTACTTGTGGCTCAGACCTGAGTGCAAGGTGGAAATAAACATCAAACATCTTTTCATTA

Primers selected for are highlighted in yellow. Underlined sequence common to both isoforms.

**Further analysis by AceView to check for alternative transcript variants has revealed that PPIA(**[**https://www.ncbi.nlm.nih.gov/IEB/Research/Acembly/av.cgi?db=human&term=PPIA&submit=Go**](https://www.ncbi.nlm.nih.gov/IEB/Research/Acembly/av.cgi?db=human&term=PPIA&submit=Go)**) contains**[**16 distinct introns**](about:blank)**(15 gt-ag, 1 gc-ag). Transcription produces**[**16 different mRNAs**](about:blank)**, 13 alternatively spliced variants and 3 unspliced forms.** **Forward and/or reverse sequence primers are found in 13 out of 16 sequences, thus highly represented.**

**>PPIA.aAug10 mRNA Sequence 2288 bp, derived from the genome**
>mRNA PPIA.aAug10, 2288 bp with coding in upper case and exons in alternate colors

gcgggcggggccgaacgtggtataaaaggggcgggaggccaggctcgtgccgttttgcagacgccaccgccgaggaaaaccgtgtactattagccATGGTCAACCCCACCGTGTTCTTCGACATTGCCGTCGACGGCGAGCCCTTGGGCCGCGTCTCCTTTGAGCTGTTTGCAGACAAGGTCCCAAAGACAGCAGAAAATTTTCGTGCTCTGAGCACTGGAGAGAAAGGATTTGGTTATAAGGGTTCCTGCTTTCACAGAATTATTCCAGGGTTTATGTGTCAGGGTGGTGACTTCACACGCCATAATGGCACTGGTGGCAAGTCCATCTATGGGGAGAAATTTGAAGATGAGAACTTCATCCTAAAGCATACGGGTCCTGGCATCTTGTCCATGGCAAATGCTGGACCCAACACAAATGGTTCCCAGTTTTTCATCTGCACTGCCAAGACTGAGTGGTTGGATGGCAAGCATGTGGTGTTTGGCAAAGTGAAAGAAGGCATGAATATTGTGGAGGCCATGGAGCGCTTTGGGTCCAGGAATGGCAAGACCAGCAAGAAGATCACCATTGCTGACTGTGGACAACTCGAATAAgtttgacttgtgttttatcttaaccaccagatcattccttctgtagctcaggagagcacccctccaccccatttgctcgcagtatcctagaatctttgtgctctcgctgcagttccctttgggttccatgttttccttgttccctcccatgcctagctggattgcagagttaagtttatgattatgaaataaaaactaaataacaattgtcctcgtttgagttaagagtgttgatgtaggctttattttaagcagtaatgggttacttctgaaacatcacttgtttgcttaattctacacagtacttagattttttttactttccagtcccaggaagtgtcaatgtttgttgagtggaatattgaaaatgtaggcagcaactgggcatggtggctcactgtctgtaatgtattacctgaggcagaagaccacctgagggtaggagtcaagatcagcctgggcaacatagtgagacgctgtctctacaaaaaataattagcctggcctggtggtgcatgcctagtcctagctgatctggaggctgacgtgggaggattgcttgagcctagagtgagctattatcatgccactgtacagcctgggtgttcacagatcttgtgtctcaaaggtaggcagaggcaggaaaagcaaggagccagaattaagaggttgggtcagtctgcagtgagttcatgcatttagaggtgttcttcaagatgactaatgtcaaaaattgagacatctgttgcggttttttttttttttttttcccctggaatgcagtggcgtgatctcagctcactgcagcctccgcctcctgggttcaagtgattctagtgcctcagcctcctgagtagctgggataatgggcgtgtgccaccatgcccagctaatttttgtatttttagtatagatggggtttcatcattttgaccaggctggtctcaaactcttgacctcagctgatgcgcctgccttggcctcccaaactgctgagattacagatgtgagccaccgcaccctacctcattttctgtaacaaagctaagcttgaacactgttgatgttcttgagggaagcatattgggctttaggctgtaggtcaagtttatacatcttaattatggtggaattcctatgtagagtctaaaaagccaggtacttggtgctacagtcagtctccctgcagagggttaaggcgcagactacctgcagtgaggaggtactgcttgtagcatatagagcctctccctagctttggttatggaggctttgaggttttgcaaacctgaccaatttaagccataagatctggtcaaagggatacccttcccactaaggacttggtttctcaggaaattatatgtacagtgcttgctggcagttagatgtcaggacaatctaagctgagaaaaccccttctctgcccaccttaacagacctctagggttcttaacccagcaatcaagtttgcctatcctagaggtggcggatttgatcatttggtgtgttgggcaatttttgttttactgtctggttccttctgcgtgaattaccaccaccaccacttgtgcatctcagtcttgtgtgttgtctggttacgtattccctgggtgataccattcaatgtcttaatgtacttgtggctcagacctgagtgcaaggtggaaataaacatcaaacatcttttcatta

**>PPIA.bAug10 mRNA Sequence 2230 bp, derived from the genome**
>mRNA PPIA.bAug10, 2230 bp with coding in upper case and exons in alternate colors

cgttaaatgagttcttaaagatcagttgtaattatagcatagtatctaaacttggcgcgtgtcttcaaagttaaatattgagtacgattccgttccagttaacatggatagaccttagggagtagcgaaataggatgttagtggttttattcctttaaatcacatctcaaaaggccaccaatggctagtttgggatcttattccgaaaatagattgatcctcatgcagtcttcgtgaggacagagcgatttccttgttgcctaccctgtccatagtgcctggcacataggcactgaaacactgcatgttaatccacaccccaccccacctatgagtgtagtcaaagctggtaagtgacaagggctttcgtggaaacttggcctgacctaatgttgggcatcaggttacccaaagagcttcagggaaatgagaaaggacttgcaggtcttgatgagaatggaggggtaactgccaatgagggctttggctttagcgaaagtctgaaagggaagccataggaacttaaacgtaccgactataaagctctgagaaaagctgatgttttagaaagaccatacattctaggtacaaatacctaaaaactaaaaaataagtacgttggccaggcgggcggatcacgaagtcaggagattgagaccgtcctggccaacatggtgaaaccccacctctattaaaaatacaaaaattagctgggcgtggtggcgcttgcctgtaatctcagctactctagaggctgaggcaggagaatcgcttgaaccccggaggcggaggctgcagtgagccgagatcgtgccactgcactccagcctggtgacagtgagactcttgtctcaaaaaaaaaaaagtacattgctataagagaagtgcacacggatactagtagttaattcagtcacatctgtgaaatagcttataaaatgctacttttaaacaagctgtttttatgaaagggcttgtaaatgtttatggtatttaagctacctctctagccataacgtattatacattcaagaaaggttcaaaaccagatatactagaaaccaatctttattttttaccccactactaggtaaggggcctggataccaagaagtgactgctcatctaatccataaagctatgttaacagattggaggtagtagcattttcattacaagtgactaaaagaacagctgtttacccctgatcgtgcagcagtgcttgctgttccttagaattttgccttgtaagttctagctcaagttggggggtggtgatagacatttaagaagccatatatcttttcagaagtaggtgtgatgtactaaaagtttgagacactttctagaagtctcactatttaagttatgactagtattggatttttggcatgtctttgggtttcatgtttcttaacccaactgcctgcagggccttatggctgtcaggagcagttcttgggaattaaagtaattactgaagaagtattctagtgagaaaatgaatttatgactcagaagcccctaaagacATGGGTACTAAGCAACAAAATAAGCAGATGTTAATTAACTGTAATTTTCTCTTACAGCTGTTTGCAGACAAGGTCCCAAAGACAGCAGAAAATTTTCGTGCTCTGAGCACTGGAGAGAAAGGATTTGGTTATAAGGGTTCCTGCTTTCACAGAATTATTCCAGGGTTTATGTGTCAGGGTGGTGACTTCACACGCCATAATGGCACTGGTGGCAAGTCCATCTATGGGGAGAAATTTGAAGATGAGAACTTCATCCTAAAGCATACGGGTCCTGGCATCTTGTCCATGGCAAATGCTGGACCCAACACAAATGGTTCCCAGTTTTTCATCTGCACTGCCAAGACTGAGTGGTTGGATGGCAAGCATGTGGTGTTTGGCAAAGTGAAAGAAGGCATGAATATTGTGGAGGCCATGGAGCGCTTTGGGTCCAGGAATGGCAAGACCAGCAAGAAGATCACCATTGCTGACTGTGGACAACTCGAATAAgtttgacttgtgttttatcttaaccaccagatcattccttctgtagctcaggagagcacccctccaccccatttgctcgcagtatcctagaatctttgtgctctcgctgcagttccctttgggttccatgttttccttgttccctcccatgcctagctggattgcagagttaagtttatgattatgaaataaaaactaaataacaattgtcc

**>PPIA.cAug10 mRNA Sequence 1347 bp, derived from the genome**
>mRNA PPIA.cAug10, 1347 bp with coding in upper case and exons in alternate colors

gttttgcagacgccaccgccgaggaaaaccgtgtactattagccATGGTCAACCCCACCGTGTTCTTCGACATTGCCGTCGACGGCGAGCCCTTGGGCCGCGTCTCCTTTGAGCTGTTTGCAGACAAGGTCCCAAAGACAGCAGAAAATTTTCGTGCTCTGAGCACTGGAGAGAAAGGATTTGGTTATAAGGGTTCCTGCTTTCACAGAATTATTCCAGGGTTTATGTGTCAGGGTGGTGACTTCACACGCCATAATGGCACTGGTGGCAAGTCCATCTATGGGGAGAAATTTGAAGATGAGAACTTCATCCTAAAGCATACGGGTCCTGGCATCTTGTCCATGGCAAATGCTGGACCCAACACAAATGGTTCCCAGTTTTTCATCTGCACTGCCAAGACTGAGTGGTAAgggtacaacatggcacactaaccacctgactaaatgaaaagttgccctggggggaacggaacaaacactacttttcttcaacctttgcttccacagactttttcatccctaagatactagaagaagagcatacataaacgacaaatatagccaatgtgatacagaatgtcagatactatgatagaaacttggcccttagctgggtggttgaattaggtgctacttttttgagatggagttttgctctgttgccaggttggagtgcagtggcacaatctgggctcactgcaacctctgcctcctgggttcaagcgattctcctgccttggcctcctgagtagctgagaatacagatgtgtgccagcatgcctggctaattttttgtatttttgtggagacggggtttcatcatgttggccaagctggtcttgaactcgtgacttaaggtgaaccacctgccttggccccccaaagtgctgggatttcaggcatgagccactgcgcccaaccaattaagtgctttttttttttttttcttttctcagactggatctcgctcttatctcccaggttggagtgcagtggtgccatctcagctcactgcaacctcctcccgggttcaagcaattcttctgcctcagcctctcaagtagctggaactacaggcatgcaccaccactcccagctaaattgtgtattattagtagagcgggatttaccatgttgtccaggctggtctcgaactcctgggctcaagtgatctgcctgccttgacccccccaaagtgctgggattacaggcatgagccactgtgcccacccaattaagtgctgcttttatgttactattaataacatgcggttggttgggttttttgtttctttggggtttttgttttgttttgtttgtttttgggggaggggggcgcaattcattc

**>PPIA.dAug10 mRNA Sequence 868 bp, derived from the genome**
>mRNA PPIA.dAug10, 868 bp with coding in upper case and exons in alternate colors

gttttgcagacgccaccgccgaggaaaaccgtgtactattagccATGGTCAACCCCACCGTGTTCTTCGACATTGCCGTCGACGGCGAGCCCTTGGGCCGCGTCTCCTTTGAGCTGTTTGCAGACAAGGTCCCAAAGACAGCAGAAAATTTTCGTGCTCTGAGCACTGGAGAGAAAGGATTTGGTTATAAGGGTTCCTGCTTTCACAGAATTATTCCAGGGTTTATGTGTCAGGGTGGTGACTTCACACGCCATAATGGCACTGGTGGCAAGTCCATCTATGGGGAGAAATTTGAAGATGAGAACTTCATCCTAAAGCATACGGGTCCTGGCATCTTGTCCATGGCAAATGCTGGACCCAACACAAATGGTTCCCAGTTTTTCATCTGCACTGCCAAGACTGAGTGActggatctcgctcttatctcccaggttggagtgcagtggtgccatctcagctcactgcaacctcctcccgggttcaagcaattcttctgcctcagcctctcaagtagctggaactacaggttggatggcaagcatgtggtgtttggcaaagtgaaagaaggcatgaatattgtggaggccatggagcgctttgggtccaggaatggcaagaccagcaagaagatcaccattgctgactgtggacaactcgaataagtttgacttgtgttttatcttaaccaccagatcattccttctgtagctcaggagagcacccctccaccccatttgctcgcagtatcctagaatctttgtgctctcgctgcagttccctttgggttccatgttttccttgttccctcccatgcctagctggattgcagagttaagtttatgattatgaaataaaaactaaataacaa

**>PPIA.eAug10 mRNA Sequence 1152 bp, derived from the genome**
>mRNA PPIA.eAug10, 1152 bp with coding in upper case and exons in alternate colors

gttttgcagacgccaccgccgaggaaaaccgtgtactattagccatggtcaaccccaccgtgttcttcgacattgccgtcgacggcgagcccttgggccgcgtctcctttgaggtcgggcgggcggcggcgtgcgggaatggggcccagaaagtgggccggggtcggggtgggtggtagcgccccaaaggcccgggcgcggggcgaccctgcttgaggggcgagcgcgggcgggctgcggcgccatttcctgacgaggggccattttgggaggtccgcgagtcgcggggaggaggccgggacggcggcggacaaaggcaggcggggcggctgcgaggccgttgggggagggggcccgcgtccgcccgcccgcctcatgtggccgcgccctgtcctgtccgacgcacgtgctcggcggccgcgctcaggtccgcgccttgagagtcgttgggctccgccctagcttggcctgggcgccgcagaccggagccagaagcacgctcgcgggggcttgcgaccgccttcctgggaagctgtcccctggcaggcatgggtgctttacatcctgagctgggaagctgtttgcttgagggtttttctcaaggatcgaggcgcggtgtgagcccgtccatgctcggtcctgtagatcccgggaggccatgttataaaaggagacttgctgggatgtgacgggttgccacttgaaatatcttccatttggataaagtaggaatatttatacATGTGCCCCAAACGTCCCTCCGTGTCCCCCACCCCCAAGCGGAAATGTGAAAATGGGCCTTGCCTTTGCTGGTGCCCAAGGACCGCCTTCCACTGCAGTGACGGCGCTGGCGGGGGAGGCGCTCTTGAGCCCCTCCCGATTGTCCCTCTGCCTAGCAAGCAAGTTGCGACTGGCCACAAGGCAGGCCTCTTCCGACCAAGCTGTTTGCAGACAAGGTCCCAAAGACAGCAGAAAATTTTCGTGCTCTGAgcactggagagaaaggatttggttataagggttcctgctttcacagaattattccagggtttatgtgtcagggtggtgacttcacacgccataatggcactggtggcaagtccatctatggggagaaatttgaagatgagaacttcatcctaaagcatacgg

**>PPIA.fAug10 mRNA Sequence 695 bp, derived from the genome**
>mRNA PPIA.fAug10, 695 bp with coding in upper case and exons in alternate colors

ttttgcagacgccaccgccgaggaaaaccgtgtactattagccATGGTCAACCCCACCGTGTTCTTCGACATTGCCGTCGACGGCGAGCCCTTGGGCCGCGTCTCCTTTGAGTGTGTCTCTATGCATGGAGTAAGAAAGAAACCATCCTACAATAGCACCAAATCCAGCATGGATGGCTGTGTTGGTGCAGGACTTTCACATGTCTTTGGAGTAGAATAAgatgtgccaatccagactcaaccccatctcatcaagatccagtgatcttcgttttccgattgtctagcccaagctctcttcctccccagactcatcctccaccctgctactggacttgtctttgtactctcaaagcagtgtgaggagattcatcaaccggtggtgtggacatgtgaacagcgtgaggcagagagttataagcttcttgagggtggcagccagacagtagcagtttgtgtgcaaagcccctgcagtggcatatggaagactttcaatacatcaggaccagaggatgagctggaaatgaaatcacactcactggtgggagaaaaccaaacttgatgacagaggaccagtaaaggaagcagcttgagtatgtgtcctcagatgcagacacaaggccttgccttcctggatgagacaggcactgactggagcagggtttgccagactcaaagggccacattttaggga

**>PPIA.gAug10 mRNA Sequence 541 bp, derived from the genome**
>mRNA PPIA.gAug10, 541 bp with coding in upper case and exons in alternate colors

ttttgcagacgccaccgccgaggaaaaccgtgtactattagccATGGTCAACCCCACCGTGTTCTTCGACATTGCCGTCGACGGCGAGCCCTTGGGCCGCGTCTCCTTTGAGCTGTTTGCAGACAAGGTCCCAAAGACAGCAGGGTGGTGActtcacacgccataatggcactggtggcaagtccatctatggggagaaatttgaagatgagaacttcatcctaaagcatacgggtcctggcatcttgtccatggcaaatgctggacccaacacaaatggttcccagtttttcatctgcactgccaagactgagtggttggatggcaagcatgtggtgtttggcaaagtgaaagaaggcatgaatattgtggaggccatggagcgctttgggtccaggaatggcaagaccagcaagaagatcaccattgctgactgtggacaactcgaataagtttgacttgtgttttatcttaaccaccagatcattccttctgtagctcaggagagcacccctccaccccatttgctcgcagtatccta

**>PPIA.hAug10 mRNA Sequence 2135 bp, derived from the genome**
>mRNA PPIA.hAug10, 2135 bp with coding in upper case and exons in alternate colors

cacctatgagtgtagtcaaagctggtaagtgacaagggctttcgtggaaacttggcctgacctaatgttgggcatcaggttacccaaagagcttcagggaaatgagaaaggacttgcaggtcttgatgagaatggaggggtaactgccaatgagggctttggctttagcgaaagtctgaaagggaagccataggaacttaaacgtaccgactataaagctctgagaaaagctgatgttttagaaagaccatacattctaggtacaaatacctaaaaactaaaaaataagtacgttggccaggcgggcggatcacgaagtcaggagattgagaccgtcctggccaacatggtgaaaccccacctctattaaaaatacaaaaattagctgggcgtggtggcgcttgcctgtaatctcagctactctagaggctgaggcaggagaatcgcttgaaccccggaggcggaggctgcagtgagccgagatcgtgccactgcactccagcctggtgacagtgagactcttgtctcaaaaaaaaaaaagtacattgctataagagaagtgcacacggatactagtagttaattcagtcacatctgtgaaatagcttataaaatgctacttttaaacaagctgtttttatgaaagggcttgtaaatgtttatggtatttaagctacctctctagccataacgtattatacattcaagaaaggttcaaaaccagatatactagaaaccaatctttattttttaccccactactaggtaaggggcctggataccaagaagtgactgctcatctaatccataaagctatgttaacagattggaggtagtagcattttcattacaagtgactaaaagaacagctgtttacccctgatcgtgcagcagtgcttgctgttccttagaattttgccttgtaagttctagctcaagttggggggtggtgatagacatttaagaagccatatatcttttcagaagtaggtgtgatgtactaaaagtttgagacactttctagaagtctcactatttaagttatgactagtattggatttttggcatgtctttgggtttcatgtttcttaacccaactgcctgcagggccttatggctgtcaggagcagttcttgggaattaaagtaattactgaagaagtattctagtgagaaaatgaatttatgactcagaagcccctaaagacATGGGTACTAAGCAACAAAATAAGCAGATGTTAATTAACTGTAATTTTCTCTTACAGCTGTTTGCAGACAAGGTCCCAAAGACAGCAGAAAATTTTCGTGCTCTGAGCACTGGAGAGAAAGGATTTGGTTATAAGGGTTCCTGCTTTCACAGAATTATTCCAGGGTTTATGTGTCAGGTACGAAATTTACTGAATTTTATTTTATTTGGGTTGCTCCCTTCATTTGGGATTGAGCCAGAATATTTCAGGATACACATATCTGAACTGTTACTCTACCATTTCGGTTCTATTTAAcccttctattcagtttgaacttgggtttaaagtttgaaccttgcagatttggcacacttcatggttatgttgtcagaagtgacatttttcctatatgttgacagggtggtgacttcacacgccataatggcactggtggcaagtccatctatggggagaaatttgaagatgagaacttcatcctaaagcatacgggtcctggcatcttgtccatggcaaatgctggacccaacacaaatggttcccagtttttcatctgcactgccaagactgagtggttggatggcaagcatgtggtgtttggcaaagtgaaagaaggcatgaatattgtggaggccatggagcgctttgggtccaggaatggcaagaccagcaagaagatcaccattgctgactgtggacaactcgaataagtttgacttgtgttttatcttaaccaccagatcattccttctgtagctcaggagagcacccctccaccccatttgctcgcagtatcctagaatctttgtgctctcgctgcagttccctttgggttccatgttttccttgttccctcccatgcctagctggattgcagagttaagtttatgattatgaaataaaaactaaataacaattgtcctcgtttgag

**>PPIA.iAug10-unspliced mRNA Sequence 1670 bp, derived from the genome**
>mRNA PPIA.iAug10-unspliced, 1670 bp with coding in upper case and exons in alternate colors

gctacttttaaacaagctgtttttatgaaagggcttgtaaatgtttatggtatttaagctacctctctagccataacgtattatacattcaagaaaggttcaaaaccagatatactagaaaccaatctttattttttaccccactactaggtaaggggcctggataccaagaagtgactgctcatctaatccataaagctatgttaacagattggaggtagtagcattttcattacaagtgactaaaagaacagctgtttacccctgatcgtgcagcagtgcttgctgttccttagaattttgccttgtaagttctagctcaagttggggggtggtgatagacatttaagaagccatatatcttttcagaagtaggtgtgatgtactaaaagtttgagacactttctagaagtctcactatttaagttATGACTAGTATTGGATTTTTGGCATGTCTTTGGGTTTCATGTTTCTTAACCCAACTGCCTGCAGGGCCTTATGGCTGTCAGGAGCAGTTCTTGGGAATTAAAGTAATTACTGAAGAAGTATTCTAGtgagaaaatgaatttatgactcagaagcccctaaagacatgggtactaagcaacaaaataagcagatgttaattaactgtaattttctcttacagctgtttgcagacaaggtcccaaagacagcaggttggtccattttctaagtttaacaaagatgttccaattgtgacagtttgtgtgtgtgtgtgtatatatatatttttatgtatgtatatatgtgtttaatttttttttaaacagaaaattttcgtgctctgagcactggagagaaaggatttggttataagggttcctgctttcacagaattattccagggtttatgtgtcaggtacgaaatttactgaattttattttatttgggttgctcccttcatttgggattgagccagaatatttcaggatacacatatctgaactgttactctaccatttcggttctatttaacccttctattcagtttgaacttgggtttaaagtttgaaccttgcagatttggcacacttcatggttatgttgtcagaagtgacatttttcctatatgttgacagggtggtgacttcacacgccataatggcactggtggcaagtccatctatggggagaaatttgaagatgagaacttcatcctaaagcatacgggtcctggcatcttgtccatggcaaatgctggacccaacacaaatggttcccagtttttcatctgcactgccaagactgagtggtaagggtacaacatggcacactaaccacctgactaaatgaaaagttgccctggggggaacggaacaaacactacttttcttcaacctttgcttccacagactttttcatccctaagatactagaagaagagcatacataaacgacaaatatagccaatgtgatacagaatgtcagatactatgatagaaacttggcccttagctgggtggttgaattaggtgctacttttttgagatggagttttgctctgttgccaggttggagtgcagtggcacaatctgggctcactgcaacctctgcctcctgggttcaagcgattctcctgccttggcctcctgagtagctgagaatacagatgtgtgccagcatgcctggctaattttttgtattt

**>PPIA.jAug10-unspliced mRNA Sequence 1167 bp, derived from the genome**
>mRNA PPIA.jAug10-unspliced, 1167 bp with coding in upper case and exons in alternate colors

cggccgcgctcaggtccgcgccttgagagtcgttgggctccgccctagcttggcctgggcgccgcagaccggagccagaagcacgctcgcgggggcttgcgaccgccttcctgggaagctgtcccctggcaggcatgggtgctttacatcctgagctgggaagctgtttgcttgagggtttttctcaaggatcgaggcgcggtgtgagcccgtccatgctcggtcctgtagatcccgggaggccatgttataaaaggagacttgctgggatgtgacgggttgccacttgaaatatcttccatttggataaagtaggaatatttatacATGTGCCCCAAACGTCCCTCCGTGTCCCCCACCCCCAAGCGGAAATGTGAAAATGGGCCTTGCCTTTGCTGGTGCCCAAGGACCGCCTTCCACTGCAGTGACGGCGCTGGCGGGGGAGGCGCTCTTGAGCCCCTCCCGATTGTCCCTCTGCCTAGCAAGCAAGTTGCGACTGGCCACAAGGCAGGCCTCTTCCGACCAAGGTGGATTACCAGTGATTACCTAATTAGTTTTGAGAGCGTTAAATGAgttcttaaagatcagttgtaattatagcatagtatctaaacttggcgcgtgtcttcaaagttaaatattgagtacgattccgttccagttaacatggatagaccttagggagtagcgaaataggatgttagtggttttattcctttaaatcacatctcaaaaggccaccaatggctagtttgggatcttattccgaaaatagattgatcctcatgcagtcttcgtgaggacagagcgatttccttgttgcctaccctgtccatagtgcctggcacataggcactgaaacactgcatgttaatccacaccccaccccacctatgagtgtagtcaaagctggtaagtgacaagggctttcgtggaaacttggcctgacctaatgttgggcatcaggttacccaaagagcttcagggaaatgagaaaggacttgcaggtcttgatgagaatggaggggtaactgccaatgagggctttggctttagcgaaagtctgaaagggaagccataggaacttaaacgtaccgactataaagctctgagaaaagctgatgttttagaaagaccatacattctaggtacaaatacctaaaaact

**>PPIA.kAug10 mRNA Sequence 935 bp, derived from the genome**
>mRNA PPIA.kAug10, 935 bp with coding in upper case and exons in alternate colors

ggccaggctcgtgccgttttgcagacgccaccgccgaggaaaaccgtgtactattagccatggtcaaccccaccgtgttcttcgacattgccgtcgacggcgagcccttgggccgcgtctcctttgaggtaaggggcctggataccaagaagtgactgctcatctaatccataaagctatgttaacagattggaggtagtagcattttcattacaagtgactaaaagaacagctgtttacccctgatcgtgcagcagtgcttgctgttccttagaattttgccttctgtttgcagacaaggtcccaaagacagcagaaaattttcgtgctctgagcactggagagaaaggatttggttataagggttcctgctttcacagaattattccagggtttATGTGTCAGGGTGGTGACTTCACACGCCATAATGGCACTGGTGGCAAGTCCATCTATGGGGAGAAATTTGAAGATGAGAACTTCATCCTAAAGCATACGGGTCCTGGCATCTTGTCCATGGCAAATGCTGGACCCAACACAAATGGTTCCCAGTTTTTCATCTGCACTGCCAAGACTGAGTGGTTGGATGGCAAGCATGTGGTGTTTGGCAAAGTGAAAGAAGGCATGAATATTGTGGAGGCCATGGAGCGCTTTGGGTCCAGGAATGGCAAGACCAGCAAGAAGATCACCATTGCTGACTGTGGACAACTCGAATAAgtttgacttgtgttttatcttaaccaccagatcattccttctgtagctcaggagagcacccctccaccccatttgctcgcagtatcctagaatctttgtgctctcgctgcagttccctttgggttccatgttttccttgttccctcccatgcctagctggattgcagagttaagtttatgattatgaaataaaaactaaataacaattgtcctcgtttgag

**>PPIA.lAug10 mRNA Sequence 835 bp, derived from the genome**
>mRNA PPIA.lAug10, 835 bp with coding in upper case and exons in alternate colors

gtagtagcattttcattacaagtgactaaaagaacagctgtttacccctgatcgtgcagcagtgcttgctgttccttagaattttgccttctgtttgcagacaaggtcccaaagacagcagaaaattttcgtgctctgagcactggagagaaaggatttggttataagggttcctgctttcacagaattattccagggtttatgtgtcagggtggtgacttcacacgccataatggcactggtggcaagtccatctatggggagaaatttgaagatgagaacttcatcctaaagcatacgggtcctggcatcttgtccatggcaaATGCTGGACCCAACACAAATGGTTCCCAGTTTTTCATCTGCACTGCCAAGACTGAGTGACTGGATCTCGCTCTTATCTCCCAGGTTGGAGTGCAGTGGTGCCATCTCAGCTCACTGCAACCTCCTCCCGGGTTCAAGCAATTCTTCTGCCTCAGCCTCTCAAGTAGCTGGAACTACAGGTTGGATGGCAAGCATGTGGTGTTTGGCAAAGTGAaagaaggcatgaatattgtggaggccatggagcgctttgggtccaggaatggcaagaccagcaagaagatcaccattgctgactgtggacaactcgaataagtttgacttgtgttttatcttaaccaccagatcattccttctgtagctcaggagagcacccctccaccccatttgctcgcagtatcctagaatctttgtgctctcgctgcagttccctttgggttccatgttttccttgttccctcccatgcctagctggattgcagagttaagtttatgattatgaaataaaaac

**>PPIA.mAug10 mRNA Sequence 828 bp, derived from the genome**
>mRNA PPIA.mAug10, 828 bp with coding in upper case and exons in alternate colors

gttttgcagacgccaccgccgaggaaaaccgtgtactattagccatggtcaaccccaccgtgttcttcgacattgccgtcgacggcgagcccttgggccgcgtctcctttgaggtaaggggcctggataccaagaagtgactgctcatctaatccataaagctatgttaacagattggagctgtttgcagacaaggtcccaaagacagcagaaaattttcgtgctctgagcactggagagaaaggatttggttataagggttcctgctttcacagaattattccagggtttATGTGTCAGGGTGGTGACTTCACACGCCATAATGGCACTGGTGGCAAGTCCATCTATGGGGAGAAATTTGAAGATGAGAACTTCATCCTAAAGCATACGGGTCCTGGCATCTTGTCCATGGCAAATGCTGGACCCAACACAAATGGTTCCCAGTTTTTCATCTGCACTGCCAAGACTGAGTGGTTGGATGGCAAGCATGTGGTGTTTGGCAAAGTGAAAGAAGGCATGAATATTGTGGAGGCCATGGAGCGCTTTGGGTCCAGGAATGGCAAGACCAGCAAGAAGATCACCATTGCTGACTGTGGACAACTCGAATAAgtttgacttgtgttttatcttaaccaccagatcattccttctgtagctcaggagagcacccctccaccccatttgctcgcagtatcctagaatctttgtgctctcgctgcagttccctttgggttccatgttttccttgttccctcccatgcctagctggattgcagagttaagtttatgattatgaaataaaaactaaataacaattgtcctcgtttg

**>PPIA.nAug10-unspliced mRNA Sequence 646 bp, derived from the genome**
>mRNA PPIA.nAug10-unspliced, 646 bp with coding in upper case and exons in alternate colors

accgtgttagccaggatggtctcgatctcctgacctcgtgatccgcccgccttggcctcccaaagtgctgggattacaggcgtgagccaccgcacccggcctatatgtgtaactctttaatggtaattggagaatcatgtttaatgacatttagtacaaaaggcttcagttaaaaaaaaaaaaaaaagctacctttctcgtcttggttcATGACACATGGAGGCTGCTTGTTTGTGGTTGCCAGTCATAGTGATTGTTCTTCCTTTTCAAGGTTGGATGGCAAGCATGTGGTGTTTGGCAAAGTGAAAGAAGGCATGAATATTGTGGAGGCCATGGAGCGCTTTGGGTCCAGGAATGGCAAGACCAGCAAGAAGATCACCATTGCTGACTGTGGACAACTCGAATAAgtttgacttgtgttttatcttaaccaccagatcattccttctgtagctcaggagagcacccctccaccccatttgctcgcagtatcctagaatctttgtgctctcgctgcagttccctttgggttccatgttttccttgttccctcccatgcctagctggattgcagagttaagtttatgattatgaaataaaaactaaataacaattgtcctcgtttgagttaagagtgttgatgtag

**>PPIA.oAug10 mRNA Sequence 584 bp, derived from the genome**
>mRNA PPIA.oAug10, 584 bp with coding in upper case and exons in alternate colors

GttttgcagacgccaccgccgaggaaaaccgtgtactattagccatggtcaaccccaccgtgttcttcgacattgccgtcgacggcgagcccttgggccgcgtctcctttgagtgacggcgctggcgggggaggcgctcttgagcccctcccgattgtccctctgcctagcaagcaagttgcgactggccacaaggcaggcctcttccgaccaagctgtttgcagacaaggtcccaaagacagcagaaaattttcgtgctctgagcactggagagaaaggatttggttataagggttcctgctttcacagaattattccagggtttATGTGTCAGGGTGGTGACTTCACACGCCATAATGGCACTGGTGGCAAGTCCATCTATGGGGAGAAATTTGAAGATGAGAACTTCATCCTAAAGCATACGGGTCCTGGCATCTTGTCCATGGCAAATGCTGGACCCAACACAAATGGTTCCCAGTTTTTCATCTGCACTGCCAAGACTGAGTGGTTGGATGGCAAGCATGTGGTGTTTGGCAAAGTGAAAGAAGGCATGAATATTGTGGAGGCCATGGAGCGCTTTGGG

**>PPIA.pAug10 mRNA Sequence 324 bp, derived from the genome**
>mRNA PPIA.pAug10, 324 bp with coding in upper case and exons in alternate colors

aTTCCAGGGTTTATGTGTCAGGTTGGATGGCAAGCATGTGGTGTTTGGCAAAGTGAAAGAAGGCATGAATATTGTGGAGGCCATGGAGCGCTTTGGGTCCAGGAATGGCAAGACCAGCAAGAAGATCACCATTGCTGACTGTGGACAACTCGAATAAgtttgacttgtgttttatcttaaccaccagatcattccttctgtagctcaggagagcacccctccaccccatttgctcgcagtatcctagaatctttgtgctctcgctgcagttccctttgggttccatgttttccttgttccctcccatgcctagctggattgcag

**Ppia mRNA SEQUENCE (Mus musculus)**

According to NCBI gene page:

[**https://www.ncbi.nlm.nih.gov/gene/268373**](https://www.ncbi.nlm.nih.gov/gene/268373)

Ppia has only 1 reference Sequences for mRNA and Protein(s):

[**NM_008907.2**](https://www.ncbi.nlm.nih.gov/nuccore/NM_008907.2)**→**[**NP_032933.1**](https://www.ncbi.nlm.nih.gov/protein/NP_032933.1)**peptidyl-prolyl cis-trans isomerase A**

**Mus musculus peptidylprolyl isomerase A (Ppia), mRNA**

NCBI Reference Sequence: NM_008907.2

CGCTTTGCAGACGCCACTGTCGCTTTTCGCCGCTTGCTGCAGCC**ATGGTCAACCCCACCGTGTTCTTCGACATCACGGCCGATGACGAGCCCTTGGGCCGCGTCTCCTTCGAGCTGTTTGCAGACAAAGTTCCAAAGACAGCAGAAAACTTTCGAGCTCTGAGCACTGGAGAGAAAGGATTTGGCTATAAGGGTTCCTCCTTTCACAGAATTATTCCAGGATTCATGTGCCAGGGTGGTGACTTTACACGCCATAATGGCACTGGCGGCAGGTCCATCTACGGAGAGAAATTTGAGGATGAGAACTTCATCCTAAAGCATACAGGTCCTGGCATCTTGTCCATGGCAAATGCTGGACCAAACACAAACGGTTCCCAGTTTTTTATCTGCACTGCCAAGACTGAATGGCTGGATGGCAAGCATGTGGTCTTTGGGAAGGTGAAAGAAGGCATGAACATTGTGGAAGCCATGGAGCGTTTTGGGTCCAGGAATGGCAAGACCAGCAAGAAGATCACCATTTCCGACTGTGGACAGCTCTAA**TTTCTTTTGACTTGCGGGCATTTTACCCATCAAACCATTCCTTCTGTAGCTCAGGAGAGCGTCCCTACCCCATCTGCTCGCAATGTCCTGTAATCTCTGCTCTCACTGAAGTTCTTTGGGTTCCATATTTTCCTCATTCCCCTTCAAGTCTAGCTGGATTGCAAAGTTAAGTTTATGATTATGAATAAAAACTAAATAAGAACTGTT

Primers selected for are highlighted in yellow.

**Further analysis by AceView to check for alternative transcript variants has revealed that Ppia(**[**https://www.ncbi.nlm.nih.gov/IEB/Research/Acembly/av.cgi?db=mouse&term=Ppia&submit=Go**](https://www.ncbi.nlm.nih.gov/IEB/Research/Acembly/av.cgi?db=mouse&term=Ppia&submit=Go)**). The gene contains**[**8 distinct introns**](about:blank)**(7 gt-ag, 1 gc-ag). Transcription produces**[**11 different mRNAs**](about:blank)**, 9 alternatively spliced variants and 2 unspliced forms. Forward and/or Reverse primers are repeated in 10 out of 11 splicing forms corresponding to the longest coding regions sequences.**

**>Ppia.aSep07 mRNA Sequence 794 bp, derived from the genome**
>mRNA Ppia.aSep07, 794 bp with coding in upper case and exons in alternate colors

CCCGAACCGGGTATAAAGGAAGCCGCGGCGAGGCTCGTGCCGCTTTGCAGACGCCACTGTCGCTTTTCGCCGCTTGCTGCAGCCATGGTCAACCCCACCGTGTTCTTCGACATCACGGCCGATGACGAGCCCTTGGGCCGCGTCTCCTTCGAGGTCGGGTGGCTGTTTGCAGACAAAGTTCCAAAGACAGCAGAAAACTTTCGAGCTCTGAGCACTGGAGAGAAAGGATTTGGCTATAAGGGTTCCTCCTTTCACAGAATTATTCCAGGATTCATGTGCCAGGGTGGTGACTTTACACGCCATAATGGCACTGGCGGCAGGTCCATCTACGGAGAGAAATTTGAGGATGAGAACTTCATCCTAAAGCATACAGGTCCTGGCATCTTGTCCATGGCAAATGCTGGACCAAACACAAACGGTTCCCAGTTTTTTATCTGCACTGCCAAGACTGAATGGCTGGATGGCAAGCTGTGGTCTTTGGGAAGGTGAAAGAAGGCATGAACATTGTGGAAGCCATGGAGCGTTTTGGGTCCAGGAATGGCAAGACCAGCAAGAAGATCACCATTTCCGACTGTGGACAGCTCTAAtttcttttgacttgcgggcattttacccatcaaaccattccttctgtagctcaggagagcgtccctaccccatctgctcgcaatgtcctgtaatctctgctctcactgaagttctttgggttccatattttcctcattccccttcaagtctagctggattgcaaagttaagtttatgattatgaataaaaactaaataagaactgt

**>Ppia.bSep07 mRNA Sequence 1170 bp, derived from the genome**
>mRNA Ppia.bSep07, 1170 bp with coding in upper case and exons in alternate colors

ggcagcgcctcaggaccccgcccgaccttgaagggccgctcgaccttgaggaccatgtttgcctcagttacttgctctgtgcaatgggaagatacgcggtaccactggggaagaccaggggccccgacgcgttcctaaggacagccagattgcgtggccaccctccctaactgccctcggactcttcccccaccgaccgcgccgcaaccatctagtaggagcccgcgggcgggaacctgcctctgcgcgctacagcgcacgcgcgcctcgcgccccgccccacccgcgcccgcgcccgcccataggcctgtcgcgccgtcactccgtttggaaagcagttgtgattgatccaggtccgggcgcgcgactcggtggtgggcggggcccgaaccgggtataaaggaagccgcggcgaggctcgtgccgctttgcagacgccactgtcgcttttcgccgcttgctgcagccATGGTCAACCCCACCGTGTTCTTCGACATCACGGCCGATGACGAGCCCTTGGGCCGCGTCTCCTTCGAGCTGTTTGCAGACAAAGTTCCAAAGACAGCAGAAAACTTTCGAGCTCTGAGCACTGGAGAGAAAGGATTTGGCTATAAGGGTTCCTCCTTTCACAGAATTATTCCAGGATTCATGTGCCAGGGTGGTGACTTTACACGCCATAATGGCACTGGCGGCAGGTCCATCTACGGAGAGAAATTTGAGGATGAGAACTTCATCCTAAAGCATACAGGTCCTGGCATCTTGTCCATGGCAAATGCTGGACCAAACACAAACGGTTCCCAGTTTTTTATCTGCACTGCCAAGACTGAATGGCTGGATGGCAAGCATGTGGTCTTTGGGAAGGTGAAAGAAGGCATGAACATTGTGGAAGCCATGGAGCGTTTTGGGTCCAGGAATGGCAAGACCAGCAAGAAGATCACCATTTCCGACTGTGGACAGCTCTAAtttcttttgacttgcgggcattttacccatcaaaccattccttctgtagctcaggagagcgtccctaccccatctgctcgcaatgtcctgtaatctctgctctcactgaagttctttgggttccatattttcctcattccccttcaagtctagctggattgcaaagttaagtttatgattatgaataaaaactaaataagaactgtt

**>Ppia.cSep07 mRNA Sequence 505 bp, derived from the genome**
>mRNA Ppia.cSep07, 505 bp with coding in upper case and exons in alternate colors

tcgcttttcgccgcttgctgcagccATGGTCAACCCCACCGTGTTCTTCGACATCACGGCCGATGACGAGCCCTTGGGCCGCGTCTCCTTCGAGCTGTTTGCAGACAAAGTTCCAAAGACAGCAGAAAACTTTCGAGCTCTGAGCACTGGAGAGAAAGGATTTGGCTATAAGGGTTCCTCCTTTCACAGAATTATTCCAGGATTCATGTGCCAGGCTGGATGGCAAGCATGTGGTCTTTGGGAAGGTGAAAGAAGGCATGAACATTGTGGAAGCCATGGAGCGTTTTGGGTCCAGGAATGGCAAGACCAGCAAGAAGATCACCATTTCCGACTGTGGACAGCTCTAATTTCTTTTGACTTGCGGGCATTTTACCCATCAAACCATTCCTTCTGTAGCTCAGGAGAGCGTCCCTACCCCATCTGCTCGCAATGTCCTGTAATCTCTGCTCTCACTGAAGTTCTTTGGGTTCCATATTTTCCTCATTCCCCTTCAAGTCTAGCTGGA

**>Ppia.dSep07 mRNA Sequence 580 bp, derived from the genome**
>mRNA Ppia.dSep07, 580 bp with coding in upper case and exons in alternate colors

GGAGGCAGAGGCAGGCGGATTTCTGAGTTCGAGGCCACGCTGGTCTACAGTCTGTTTGCAGACAAAGTTCCAAAGACAGCAGAAAACTTTCGAGCTCTGAGCACTGGAGAGAAAGGATTTGGCTATAAGGGTTCCTCCTTTCACAGAATTATTCCAGGATTCATGTGCCAGGGTGGTGACTTTACACGCCATAATGGCACTGGCGGCAGGTCCATCTACGGAGAGAAATTTGAGGATGAGAACTTCATCCTAAAGCATACAGGTCCTGGCATCTTGTCCATGGCAAATGCTGGACCAAACACAAACGGTTCCCAGTTTTTTATCTGCACTGCCAAGACTGAATGGCTGGTGGCAAGCATGTGGTCTTTGGGAAGGTGAAAGAAGGCATGAACATTGTGGAAGCCATGGAGCGTTTTGGGTCCAGGAATGGCAAGACCAGCAAGAAGATCACCATTTCCGACTGTGGACAGCTCTAAtttcttttgacttgcgggcattttacccatcaaaccattccttctgtagctcaggagagcgtccctaccccatctgctcgcaatgtcctgtaatctctgctct

**>Ppia.eSep07 mRNA Sequence 824 bp, derived from the genome**
>mRNA Ppia.eSep07, 824 bp with coding in upper case and exons in alternate colors

gacgccactgtcgcttttcgccgcttgctgcagccatggtcaaccccaccgtgttcttcgacatcacggccgatgacgagcccttgggccgcgtctccttcgagctgtttgcagacaaagttccaaagacagcaggttggtttcattttctagttttaacaagttttgtgggtaaaactataaaaaagaataagtatatgtgtatacATGTTTTTTGTTTTTTTAACAGAAAACTTTCGAGCTCTGAGCACTGGAGAGAAAGGATTTGGCTATAAGGGTTCCTCCTTTCACAGAATTATTCCAGGATTCATGTGCCAGGGTGGTGACTTTACACGCCATAATGGCACTGGCGGCAGGTCCATCTACGGAGAGAAATTTGAGGATGAGAACTTCATCCTAAAGCATACAGGTCCTGGCATCTTGTCCATGGCAAATGCTGGACCAAACACAAACGGTTCCCAGTTTTTTATCTGCACTGCCAAGACTGAATGGCTGGATGGCAAGCATGTGGTCTTTGGGAAGGTGAAAGAAGGCATGAACATTGTGGAAGCCATGGAGCGTTTTGGGTCCAGGAATGGCAAGACCAGCAAGAAGATCACCATTTCCGACTGTGGACAGCTCTAAtttcttttgacttgcgggcattttacccatcaaaccattccttctgtagctcaggagagcgtccctaccccatctg

ctcgcaatgtcctgtaatctctgctctcactgaagttctttgggttccatattttcctcattccccttcaagtctagctggattgcaaagttaagtttatgattatgaataaaaactaaataag

**>Ppia.fSep07 mRNA Sequence 406 bp, derived from the genome**
>mRNA Ppia.fSep07, 406 bp with coding in upper case and exons in alternate colors

tgtcgcttttcgccgcttgctgcagccATGGTCAACCCCACCGTGTTCTTCGACATCACGGCCGATGACGAGCCCTTGGGCCGCGTCTCCTTCGAGCTGTTTGCAGACAAAGTTCCAAAGACAGCAGAAAACTTTCGAGCTCTGAGCACTGGAGAGAAAGGATTTGGCTATAAGGGTTCCTCCTTTCACAGAATTATTCCAGGATTCATGTGCCAGGGTGGTGACTTTACACGCCATAATGGCACTGGCGGCAGGTCCATCTACGGAGAGAAATTTGAGGATGAGAACTTCATCCTAAAGCATACAGGTCCTGGCATCTTGTCCATGGCAAATGCTGGACCAAACACAAACGGTTCCCAGTTTTTTATCTGCACTGCCAAGACTGAATGGTAAgaaacagtgaatg

**>Ppia.gSep07-unspliced mRNA Sequence 509 bp, derived from the genome**
>mRNA Ppia.gSep07-unspliced, 509 bp with coding in upper case and exons in alternate colors

ggatctgttgacaagagtgcaaagccccctgctggggttcttgcttcagtgttcctttcattccctctcaagatagtggaaattgagccagtaagagtgctgtgtcggtagccatttttatctttagtgtttgatctacccctggttgctgATGGTGGTTATCATTCTTCTACAGGCTGGATGGCAAGCATGTGGTCTTTGGGAAGGTGAAAGAAGGCATGAACATTGTGGAAGCCATGGAGCGTTTTGGGTCCAGGAATGGCAAGACCAGCAAGAAGATCACCATTTCCGACTGTGGACAGCTCTAATTTCTTTTGACTTGCGGGCATTTTACCCATCAAACCATTCCTTCTGTAGCTCAGGAGAGCGTCCCTACCCCATCTGCTCGCAATGTCCTGTAATCTCTGCTCTCACTGAAGTTCTTTGGGTTCCATATTTTCCTCATTCCCCTTCAAGTCTAGCTGGATTGCAAAGTTAAgtttatgattatgaataaaaactaaataaga

**>Ppia.hSep07 mRNA Sequence 3499 bp, derived from the genome**
>mRNA Ppia.hSep07, 3499 bp with coding in upper case and exons in alternate colors

gtttaaatagcttcctcctggagaaaacacaagatatatttggaaagtaggacatttaaaaagtatttcataatctttaaaatgagggggctgcttgtgcccgttccccagagtttaagaaaacggaggcgctcacagggacctatcaggaagcggttgccattgcacaatgacggtgtgggtcctggatctaggcccagggatgcctgcctgttcgagtttccgcagagagggcagcgcctcaggaccccgcccgaccttgaagggccgctcgaccttgaggaccatgtttgcctcagttacttgctctgtgcaatgggaagatacgcggtaccactggggaagaccaggggccccgacgcgttcctaaggacagccagattgcgtggccaccctccctaactgccctcggactcttcccccaccgaccgcgccgcaaccatctagtaggagcccgcgggcgggaacctgcctctgcgcgctacagcgcacgcgcgcctcgcgccccgccccacccgcgcccgcgcccgcccataggcctgtcgcgccgtcactccgtttggaaagcagttgtgattgatccaggtccgggcgcgcgactcggtggtgggcggggcccgaaccgggtataaaggaagccgcggcgaggctcgtgccgctttgcagacgccactgtcgcttttcgccgcttgctgcagccatggtcaaccccaccgtgttcttcgacatcacggccgatgacgagcccttgggccgcgtctccttcgaggtcgggtgggcagtggtgctccagggtggggtcccgggagctgtggagggggaggctctctcgggcgcggcccgaccctccggtggagcgagcgcgggtggcggcgccatttcctgacgaggggccattttgggaggctggcgaggcgcggggaggctgggcgggctgcggacaaagccgggcggggcgcggccgttgggggagggggccgtccccgcccgtcccggcctccccgcaggcgcacgtggctgcgtcctgtggggccccgcgaggccgggcgcacgtgctccgcgcctctcccttctcccagccgttgggccccgccctgctccgctgcggcgctgcagccccgcgggcctcttcccagcctgctttccggagccccacagccttttactcggatctgagcagggaccctgcctaaggtctcaggggcgaaggctcggagagcccggacgtgctcgttagcccggttaaacctagtggtttcctaaacctagtggttcctacgaagaatataaaataatcgttttatagcagacttgagtgtgaattgaacctttggccatttggtccgaagtagccacatttggttagggaggctcaaaatacccctccgcgtcccccacccccgagcggaaacgagaaaggcttggcttttgctggtgcccaaggaccgcctcctgtcgcagtgatggcgcagcgggggaggcgctctgaacccctccctattaccctcagtgagacaggcatgttatgactggccacgctgcagtttgcattgaccaaagtggattgccagtagttacctacttaattagttctggaggttaagtggctgcttaaagatcagttagtaattagagtaccctaaacaattggcgtttttcttttacaaaattaaatattcagccgggcgtggtggcgcacgcctttaatcccagcacccgggaggcagaggcaggcggatttctgagttcgaggccacgctggtctacagtgtgagttccaggacagccagggctatacagagaaaccctgtctcgaaaaaaacaaaaaaccccaaaattaaatattattgttagcctcagtactagcccttaggccatagtggaatatgttacttagtgtctgtactgttaaactccagaagctatcattggtgagtttttaactcaaaagctagatctcccgattgcccgcctgccacagtgctgggtacacatagtaggtaggcacataaaaagagcactaggttctaattagccctcacccagcatgtatctagtcaagagctgataattgacaaggactcttgtggaaacttggcctaagcttagcctgggtaggatagtatcaggttaaccagagcttaatgggaatgagatgagcccataggtgaaaatagaggtggagctgccaagaaaggctttgaagtcataatttgatgaaaaaaaatgtggacttggaaagctgttaagctgtgagaaaaaaaaaacagtccaaaggagtcacatatttaactacttaaaccataaatgtattcctccaagagaagtgtagatgctgcaactgatgagttgtggttttcaactagctatgaaagggcatgtagatattgaagtgttggaatcctctacagtcctctacaattaggttaggagccactcacctgatgcttaaagctatgctgacaggttgaaggtggtagcattttcattgccagaaactaacagtttatttgcctaatcatgcattgtgtgacagtgctttgagaatttgattttaggtttcaacatgaagttaggagtgatggtatgcttttgagaagcctcaagtctattcagaagtaggtgtgatgtattaaaagctcttgagtcatgcttaaggagtctgtcactattttaagtaatgactagttccagatttccttcatattttttggttctcaaccctgtagaatcttaaagctgataggtgtgatttcttactaggaattgaacgttattaccaaagtggtgttctgagaatgaatttgactcacaagtccctaaagctaaagacattaagcatgagactgttcacctatatattaactagctgttcatttctttgttacagctgtttgcagacaaagttccaaagacagcagaaaactttcgagctctgagcactggagagaaaggatttggctataagggttcctcctttcacagaattattccaggattcATGTGCCAGGGTGGTGACTTTACACGCCATAATGGCACTGGCGGCAGGTCCATCTACGGAGAGAAATTTGAGGATGAGAACTTCATCCTAAAGCATACAGGTCCTGGCATCTTGTCCATGGCAAATGCTGGACCAAACACAAACGGTTCCCAGTTTTTTATCTGCACTGCCAAGACTGAATGGCTGGATGGCAAGCATGTGGTCTTTGGGAAGGTGAAAGAAGGCATGAACATTGTGGAAGCCATGGAGCGTTTTGGGTCCAGGAATGGCAAGACCAGCAAGAAGATCACCATTTCCGACTGTGGACAGCTCTAAtttcttttgacttgcgggcattttacccatcaaaccattccttctgtagctcaggagagcgtccctaccccatctgctcgcaatgtcctgtaatctctgctctcactgaagttctttgggttccatattttcctcattccccttcaagtctagctggattgcaaagttaagtttatgattatg

**>Ppia.iSep07 mRNA Sequence 757 bp, derived from the genome**
>mRNA Ppia.iSep07, 757 bp with coding in upper case and exons in alternate colors

cgtgccgctttgcagacgccactgtcgcttttcgccgcttgctgcagccatggtcaaccccaccgtgttcttcgacatcacggccgatgacgagcccttgggccgcgtctccttcgaggtcggctgtttgcagacaaagttccaaagacagcagaaaactttcgagctctgagcactggagagaaaggatttggctataagggttcctcctttcacagaattattccaggattcATGTGCCAGGGTGGTGACTTTACACGCCATAATGGCACTGGCGGCAGGTCCATCTACGGAGAGAAATTTGAGGATGAGAACTTCATCCTAAAGCATACAGGTCCTGGCATCTTGTCCATGGCAAATGCTGGACCAAACACAAACGGTTCCCAGTTTTTTATCTGCACTGCCAAGACTGAATGGCTGGATGGCAAGCATGTGGTCTTTGGGAAGGTGAAAGAAGGCATGAACATTGTGGAAGCCATGGAGCGTTTTGGGTCCAGGAATGGCAAGACCAGCAAGAAGATCACCATTTCCGACTGTGGACAGCTCTAAtttcttttgacttgcgggcattttacccatcaaaccattccttctgtagctcaggagagcgtccctaccccatctgctcgcaatgtcctgtaatctctgctctcactgaagttctttgggttccatattttcctcattccccttcaagtctagctggattgcaaagttaagtttatgattatgaataaaaactaaataagaactgttt

**>Ppia.jSep07 mRNA Sequence 1181 bp, derived from the genome**
>mRNA Ppia.jSep07, 1181 bp with coding in upper case and exons in alternate colors

cgCTTTGCAGACGCCACTGTCGCTTTTCGCCGCTTGCTGCAGCCATGGTCAACCCCACCGTGTTCTTCGACATCACGGCCGATGACGAGCCCTTGGGCCGCGTCTCCTTCGAGCTGTTTGCAGACAAAGTTCCAAAGACAGCAGAAAACTTTCGAGCTCTGAGCACTGGAGAGAAAGGATTTGGCTATAAGGGTTCCTCCTTTCACAGAATTATTCCAGGATTCATGTGCCAGGTATTTATTAAATTGAAATTTTTGTTTGTTTATTTATTTATTTTTAAAATATTTATTGATTGAttgattatatgtaagtacactgtagctttcttcagaagagagcatcagattttgttacggatggttgtgagctaccatgtggtttctgggatttgaactcaggacctttggaagagcagtcagcgctcttaaccgctgagccatctcaccagccctgaaattttttattttttagctccttttattgagccagcatgttttagttgtacttgagctagtattacaccatttctttccagttgcctgttttttcttgtgtgtgtacatagcaatacatgtgagttcttctgaggcggatgactgtccccagcacaggctctgttctttgcagtattatgtcatgcaggttggccttaaactccttatccacttgcctcttcctgtgtgctgggatttcaaatgtgttaacgtaccaaccttctaaaaaaccgctatcttgggttgggtgtgagtcttatgaagtgcctgctacatagggatcccaaaactgaggacctgagtcctagagctcctataaaaagctgggtgcggcagcacatgctatcatgtcaaccagcctagctaaagctttgagccccaggctaaagaaaaggtgggtgaaaaagttggagagcagtgcagatatactgtgccatatgtatatacacagcaagaaaatttgaaatggcataaaaactgactttgcagacttttcagttacattgtcaaagtgacattactatctgatttttacagggtggtgactttacacgccataatggcactggcggcaggtccatctacggagagaaatttgaggatgagaacttcatcctaaagcatacaggtcctggcatcttgtccatggcaaatgctggaccaaacacaaacggttcccagtttttt

**>Ppia.kSep07-unspliced mRNA Sequence 1812 bp, derived from the genome**
>mRNA Ppia.kSep07-unspliced, 1812 bp with coding in upper case and exons in alternate colors

aaaaaacaaaaaaccccaaaattaaatattattgttagcctcagtactagcccttaggccatagtggaatatgttacttagtgtctgtactgttaaactccagaagctatcattggtgagtttttaactcaaaagctagatctcccgattgcccgcctgccacagtgctgggtacacatagtaggtaggcacataaaaagagcactaggttctaattagccctcacccagcatgtatctagtcaagagctgataattgacaaggactcttgtggaaacttggcctaagcttagcctgggtaggatagtatcaggttaaccagagcttaatgggaatgagatgagcccataggtgaaaatagaggtggagctgccaagaaaggctttgaagtcataatttgatgaaaaaaaatgtggacttggaaagctgttaagctgtgagaaaaaaaaaacagtccaaaggagtcacatatttaactacttaaaccataaatgtattcctccaagagaagtgtagatgctgcaactgatgagttgtggttttcaactagctatgaaagggcatgtagatattgaagtgttggaatcctctacagtcctctacaattaggttaggagccactcacctgatgcttaaagctatgctgacaggttgaaggtggtagcattttcattgccagaaactaacagtttatttgcctaatcatgcattgtgtgacagtgctttgagaatttgattttaggtttcaacatgaagttaggagtgatggtatgcttttgagaagcctcaagtctattcagaagtaggtgtgatgtattaaaagctcttgagtcatgcttaaggagtctgtcactattttaagtaatgactagttccagatttccttcatattttttggttctcaaccctgtagaatcttaaagctgataggtgtgatttcttactaggaattgaacgttattaccaaagtggtgttctgagaatgaatttgactcacaagtccctaaagctaaagacattaagcatgagactgttcacctatatattaactagctgttcatttctttgttacagctgtttgcagacaaagttccaaagacagcaggttggtttcattttctagttttaacaagttttgtgggtaaaactataaaaaagaataagtatatgtgtatacATGTTTTTTGTTTTTTTAACAGAAAACTTTCGAGCTCTGAGCACTGGAGAGAAAGGATTTGGCTATAAGGGTTCCTCCTTTCACAGAATTATTCCAGGATTCATGTGCCAGGTATTTATTAAATTGAAATTTTTGTTTGTTTATTTATTTATTTTTAAAATATTTATTGATTGAttgattatatgtaagtacactgtagctttcttcagaagagagcatcagattttgttacggatggttgtgagctaccatgtggtttctgggatttgaactcaggacctttggaagagcagtcagcgctcttaaccgctgagccatctcaccagccctgaaattttttattttttagctccttttattgagccagcatgttttagttgtacttgagctagtattacaccatttctttccagttgcctgttttttcttgtgtgtgtacatagcaatacatgtgagttcttctgaggcggatgactgtccccagcacaggctctgttctttgcagtattatgtcatgcaggttggccttaaactccttatccacttgcctcttcctgtgtgctgggatttcaaatgtgttaacgtaccaaccttctaaaaaaccgctatcttgggttgggtgtgagtcttatgaa

***RPL19***

**RPL19 mRNA SEQUENCE (Homo sapiens)**

According to NCBI gene page:

[**https://www.ncbi.nlm.nih.gov/gene/6143#gene-expression**](https://www.ncbi.nlm.nih.gov/gene/6143#gene-expression)

RPL19 has 2 reference Sequences for mRNA and Protein(s):

[**NM_000981.4**](https://www.ncbi.nlm.nih.gov/nuccore/NM_000981.4)**→**[**NP_000972.1**](https://www.ncbi.nlm.nih.gov/protein/NP_000972.1)**60S ribosomal protein L19 isoform 1**

[**NM_001330200.1**](https://www.ncbi.nlm.nih.gov/nuccore/NM_001330200.1)**→**[**NP_001317129.1**](https://www.ncbi.nlm.nih.gov/protein/NP_001317129.1)**60S ribosomal protein L19 isoform 2**

**Using EMBOSS Needle Tool:**

[**https://www.ebi.ac.uk/Tools/services/web/toolresult.ebi?jobId=emboss_needle-I20221004-094818-0297-29231689-p1m**](https://www.ebi.ac.uk/Tools/services/web/toolresult.ebi?jobId=emboss_needle-I20221004-094818-0297-29231689-p1m)

**Pairwise sequence alignement has been performed and common sequence for both isoforms is a s follows:**

########################################

# Program: needle

# Rundate: Tue 4 Oct 2022 09:46:00

# Commandline: needle

# -auto

# -stdout

# -asequence emboss_needle-I20221004-094818-0297-29231689-p1m.asequence

# -bsequence emboss_needle-I20221004-094818-0297-29231689-p1m.bsequence

# -datafile EDNAFULL

# -gapopen 10.0

# -gapextend 0.5

# -endopen 10.0

# -endextend 0.5

# -aformat3 pair

# -snucleotide1

# -snucleotide2

# Align_format: pair

# Report_file: stdout

########################################

#=======================================

#

# Aligned_sequences: 2

# 1: NM_000981.4

# 2: NM_001330200.1

# Matrix: EDNAFULL

# Gap_penalty: 10.0

# Extend_penalty: 0.5

#

# Length: 1097

# Identity: 723/1097 (65.9%)

# Similarity: 723/1097 (65.9%)

# Gaps: 360/1097 (32.8%)

# Score: 3439.0

#

#

#=======================================

NM_000981.4 1 -------------------------------------------------- 0

NM_001330200. 1 TTTCGCTGCTGCGGCCGCAGCCATGAGGTGAGGGCGAGCTGGTCTCCATC 50

NM_000981.4 1 -------------------------------------------------- 0

NM_001330200. 51 AGGCGCTGACGCGTGTCGACAAGGGACTGTCGGTCTTGGGACCGCAGCTG 100

NM_000981.4 1 -------------------------------------------------- 0

NM_001330200. 101 GGGTTGGGGGAGATGAAATGGAGGCCGCCCTAAAGCGGCCGGTCCCGGGG 150

NM_000981.4 1 -------------------------------------------------- 0

NM_001330200. 151 TTTGGGGTAGGCCGGAGCACTTTCGTCCCGGGCCTCCGGAGTGAGGGGGG 200

NM_000981.4 1 -------------------------------------------------- 0

NM_001330200. 201 GCGGGGAGCGTCGCAGCAACTGAGACCAGGAAAAGTCTGCCCCGGCTGGT 250

NM_000981.4 1 -----------------------------------GCAGA-TAATGGGAG 14

||||| .||..||||

NM_001330200. 251 GCCGCACCGCACACGTGTCCGGTCGACCCACGCGAGCAGAGCAAACGGAG 300

NM_000981.4 15 -----------GAGCC--GGGCCC--------------------GAGC-G 30

.|||| |||||| |||| |

NM_001330200. 301 CGAACAAGACCAAGCCGTGGGCCCTTTCTTGCTTGGCACACCCGGAGCGG 350

NM_000981.4 31 AGC---TCTTTCCTTTCGC-TGCTG-----------------CGGCCGCA 59

||| |||.|.|||||.| ||.|| .||||.||

NM_001330200. 351 AGCCGATCTCTGCTTTCACGTGATGTAGGGCAAGCCTAGTGTAGGCCCCA 400

NM_000981.4 60 GCCAT-------------GAGTATGCTCAGGCTTCAGAAGAGGCTCGCCT 96

|.|.| ||||||||||||||||||||||||||||||||

NM_001330200. 401 GGCCTCCGACTGCCGAGAGAGTATGCTCAGGCTTCAGAAGAGGCTCGCCT 450

NM_000981.4 97 CTAGTGTCCTCCGCTGTGGCAAGAAGAAGGTCTGGTTAGACCCCAATGAG 146

||||||||||||||||||||||||||||||||||||||||||||||||||

NM_001330200. 451 CTAGTGTCCTCCGCTGTGGCAAGAAGAAGGTCTGGTTAGACCCCAATGAG 500

NM_000981.4 147 ACCAATGAAATCGCCAATGCCAACTCCCGTCAGCAGATCCGGAAGCTCAT 196

||||||||||||||||||||||||||||||||||||||||||||||||||

NM_001330200. 501 ACCAATGAAATCGCCAATGCCAACTCCCGTCAGCAGATCCGGAAGCTCAT 550

NM_000981.4 197 CAAAGATGGGCTGATCATCCGCAAGCCTGTGACGGTCCATTCCCGGGCTC 246

||||||||||||||||||||||||||||||||||||||||||||||||||

NM_001330200. 551 CAAAGATGGGCTGATCATCCGCAAGCCTGTGACGGTCCATTCCCGGGCTC 600

NM_000981.4 247 GATGCCGGAAAAACACCTTGGCCCGCCGGAAGGGCAGGCACATGGGCATA 296

||||||||||||||||||||||||||||||||||||||||||||||||||

NM_001330200. 601 GATGCCGGAAAAACACCTTGGCCCGCCGGAAGGGCAGGCACATGGGCATA 650

NM_000981.4 297 GGTAAGCGGAAGGGTACAGCCAATGCCCGAATGCCAGAGAAGGTCACATG 346

||||||||||||||||||||||||||||||||||||||||||||||||||

NM_001330200. 651 GGTAAGCGGAAGGGTACAGCCAATGCCCGAATGCCAGAGAAGGTCACATG 700

NM_000981.4 347 GATGAGGAGAATGAGGATTTTGCGCCGGCTGCTCAGAAGATACCGTGAAT 396

||||||||||||||||||||||||||||||||||||||||||||||||||

NM_001330200. 701 GATGAGGAGAATGAGGATTTTGCGCCGGCTGCTCAGAAGATACCGTGAAT 750

NM_000981.4 397 CTAAGAAGATCGATCGCCACATGTATCACAGCCTGTACCTGAAGGTGAAG 446

||||||||||||||||||||||||||||||||||||||||||||||||||

NM_001330200. 751 CTAAGAAGATCGATCGCCACATGTATCACAGCCTGTACCTGAAGGTGAAG 800

NM_000981.4 447 GGGAATGTGTTCAAAAACAAGCGGATTCTCATGGAACACATCCACAAGCT 496

||||||||||||||||||||||||||||||||||||||||||||||||||

NM_001330200. 801 GGGAATGTGTTCAAAAACAAGCGGATTCTCATGGAACACATCCACAAGCT 850

NM_000981.4 497 GAAGGCAGACAAGGCCCGCAAGAAGCTCCTGGCTGACCAGGCTGAGGCCC 546

||||||||||||||||||||||||||||||||||||||||||||||||||

NM_001330200. 851 GAAGGCAGACAAGGCCCGCAAGAAGCTCCTGGCTGACCAGGCTGAGGCCC 900

NM_000981.4 547 GCAGGTCTAAGACCAAGGAAGCACGCAAGCGCCGTGAAGAGCGCCTCCAG 596

||||||||||||||||||||||||||||||||||||||||||||||||||

NM_001330200. 901 GCAGGTCTAAGACCAAGGAAGCACGCAAGCGCCGTGAAGAGCGCCTCCAG 950

NM_000981.4 597 GCCAAGAAGGAGGAGATCATCAAGACTTTATCCAAGGAGGAAGAGACCAA 646

||||||||||||||||||||||||||||||||||||||||||||||||||

NM_001330200. 951 GCCAAGAAGGAGGAGATCATCAAGACTTTATCCAAGGAGGAAGAGACCAA 1000

NM_000981.4 647 GAAATAAAACCTCCCACTTTGTCTGTACATACTGGCCTCTGTGATTACAT 696

||||||||||||||||||||||||||||||||||||||||||||||||||

NM_001330200. 1001 GAAATAAAACCTCCCACTTTGTCTGTACATACTGGCCTCTGTGATTACAT 1050

NM_000981.4 697 AGATCAGCCATTAAAATAAAACAAGCCTTAATCTGCCTTCC------ 737

|||||||||||||||||||||||||||||||||||||||..

NM_001330200. 1051 AGATCAGCCATTAAAATAAAACAAGCCTTAATCTGCCTTAAAAAAAA 1097

**Homo sapiens ribosomal protein L19 (RPL19), transcript variant 1, mRNA**

NCBI Reference Sequence: NM_000981.3

GCAGATAATGGGAGGAGCCGGGCCCGAGCGAGCTCTTTCCTTTCGCTGCTGCGGCCGCAGCC**ATGAGTATGCTCAGGCTTCAGAAGAGGCTCGCCTCTAGTGTCCTCCGCTGTGGCAAGAAGAAGGTCTGGTTAGACCCCAATGAGACCAATGAAATCGCCAATGCCAACTCCCGTCAGCAGATCCGGAAGCTCATCAAAGATGGGCTGATCATCCGCAAGCCTGTGACGGTCCATTCCCGGGCTCGATGCCGGAAAAACACCTTGGCCCGCCGGAAGGGCAGGCACATGGGCATAGGTAAGCGGAAGGGTACAGCCAATGCCCGAATGCCAGAGAAGGTCACATGGATGAGGAGAATGAGGATTTTGCGCCGGCTGCTCAGAAGATACCGTGAATCTAAGAAGATCGATCGCCACATGTATCACAGCCTGTACCTGAAGGTGAAGGGGAATGTGTTCAAAAACAAGCGGATTCTCATGGAACACATCCACAAGCTGAAGGCAGACAAGGCCCGCAAGAAGCTCCTGGCTGACCAGGCTGAGGCCCGCAGGTCTAAGACCAAGGAAGCACGCAAGCGCCGTGAAGAGCGCCTCCAGGCCAAGAAGGAGGAGATCATCAAGACTTTATCCAAGGAGGAAGAGACCAAGAAATAA**AACCTCCCACTTTGTCTGTACATACTGGCCTCTGTGATTACATAGATCAGCCATTAAAATAAAACAAGCCTTAATCTGCAAAAAAAAAAAAAAAA

Primers selected for are highlighted in pink. Underlined sequence common to both isoforms.

**Further analysis by AceView to check for alternative transcript variants has revealed that RPL19 (**[**https://www.ncbi.nlm.nih.gov/IEB/Research/Acembly/av.cgi?db=human&q=RPL19**](https://www.ncbi.nlm.nih.gov/IEB/Research/Acembly/av.cgi?db=human&q=RPL19)**)**

**The gene contains**[**10 distinct introns**](about:blank)**(9 gt-ag, 1 gc-ag). Transcription produces**[**9 different mRNAs**](about:blank)**, 8 alternatively spliced variants and 1 unspliced form. This unspliced form appears not to encode a good protein. Forward and reverse sequence primers are found in six out of eight coding sequences, thus highly represented.**

**>RPL19.aAug10 mRNA Sequence 852 bp, derived from the genome**
>mRNA RPL19.aAug10, 852 bp with coding in upper case and exons in alternate colors

AAGCGGCCGGTCCCGGGGTTTGGGGTAGGCCGGAGCACTTTCGTCCCGGGCCTCCGGAGTGAGGGGGGGCGGGGAGCGTCGCAGCAACTGAGACCAGGAAAAGTCTGCCCCGGCTGGTGCCGCACCGCACACGTGTCCGGTCGACCCACGCGAGCAGAGCAAACGGAGCGAACAAGACCAAGCCGTGGGCCCTTTCTTGCTTGGCACACCCGGAGCGGAGCCGATCTCTGCTTTCACGTGATTATGCTCAGGCTTCAGAAGAGGCTCGCCTCTAGTGTCCTCCGCTGTGGCAAGAAGAAGGTCTGGTTAGACCCCAATGAGACCAATGAAATCGCCAATGCCAACTCCCGTCAGCAGATCCGGAAGCTCATCAAAGATGGGCTGATCATCCGCAAGCCTGTGACGGTCCATTCCCGGGCTCGATGCCGGAAAAACACCTTGGCCCGCCGGAAGGGCAGGCACATGGGCATAGGTAAGCGGAAGGGTACAGCCAATGCCCGAATGCCAGAGAAGGTCACATGGATGAGGAGAATGAGGATTTTGCGCCGGCTGCTCAGAAGATACCGTGAATCTAAGAAGATCGATCGCCACATGTATCACAGCCTGTACCTGAAGGTGAAGGGGAATGTGTTCAAAAACAAGCGGATTCTCATGGAACACATCCACAAGCTGAAGGCAGACAAGGCCCGCAAGAAGCTCC

TGGCTGACCAGGCTGAGGCCCGCAGGTCTAAGACCAAGGAAGCACGCAAGCGCCGTGAAGAGCGCCTCCAGGCCAAGAAGGAGGAGATCATCAAGACTTTATCCAAGGAGGAAGAGACCAAGAAATAAaacctcccactttgtctgtacata

**>RPL19.bAug10 mRNA Sequence 1587 bp, derived from the genome**
>mRNA RPL19.bAug10, 1587 bp with coding in upper case and exons in alternate colors

cagataatgggaggagccgggcccgagcgagctctttcctttcgctgctgcggccgcagccatgaggtgagggcgagctggtctccatcaggcgctgacgcgtgtcgacaagggactgtcggtcttgggaccgcagctggggttgggggagatgaaatggaggccgccctaaagcggccggtcccggggtttggggtaggccggagcactttcgtcccgggcctccggagtgagggggggcggggagcgtcgcagcaactgagaccaggaaaagtctgccccggctggtgccgcaccgcacacgtgtccggtcgacccacgcgagcagagcaaacggagcgaacaagaccaagccgtgggccctttcttgcttggcacacccggagcggagccgatctctgctttcacgtgatgtagggcaagcctagtgtaggccccaggcctccgactgccgagagaggtgatctctaactcttgactccattcactcctttggcctctcataaaggaaatctctgcgaatagccgaacgaggcttgttactgtgataaaacagggaaataagcccagaaaacagagtaacttgcctgcattcctagactagaaatcaggtctactcacctcgaatattctttaaacgctgagtaccagaaatggcataacccccctattcaatccaataagtccttggcttgacttt

ccagaggagaaatgcgaacatgaggctccgagaggtgaaggcatagcgtgggttttgaagtcttaaacccaagggggccagctgcatagcccagagccttaaagatgatttagggaagagtcttatttcgcggctgtggtgtgggtcacaaagggcaggtcttgATGGGGACGTTCATTCTTGCCCAGGATTGGCTTTCAGAGTCTAATCATGTTTTCTGTGTGTCTAGTATGCTCAGGCTTCAGAAGAGGCTCGCCTCTAGTGTCCTCCGCTGTGGCAAGAAGAAGGTCTGGTTAGACCCCAATGAGACCAATGAAATCGCCAATGCCAACTCCCGTCAGCAGATCCGGAAGCTCATCAAAGATGGGCTGATCATCCGCAAGCCTGTGACGGTCCATTCCCGGGCTCGATGCCGGAAAAACACCTTGGCCCGCCGGAAGGGCAGGCACATGGGCATAGGTAAGCGGAAGGGTACAGCCAATGCCCGAATGCCAGAGAAGGTCACATGGATGAGGAGAATGAGGATTTTGCGCCGGCTGCTCAGAAGATACCGTGAATCTAAGAAGATCGATCGCCACATGTATCACAGCCTGTACCTGAAGGTGAAGGGGAATGTGTTCAAAAACAAGCGGATTCTCATGGAACACATCCACAAGCTGAAGGCAGACAAGGCCCGCAAGAAGCTCCTGGCGCTGAGGCC

CGCAGGTCTAAGACCAAGGAAGCACGCAAGCGCCGTGAAGAGCGCCTCCAGGCCAAGAAGGAGGAGATCATCAAGACTTTATCCAAGGAGGAAGAGACCAAGAAATAAAACCTCCCACTTTGTCTGTACATACTGGCCTCTGTGAttacatagatcagccattaaaataaaacaagccttaatctgc

**>RPL19.cAug10 mRNA Sequence 736 bp, derived from the genome**
>mRNA RPL19.cAug10, 736 bp with coding in upper case and exons in alternate colors

cagataatgggaggagccgggcccgagcgagctctttcctttcgctgctgcggccgcagccATGAGTATGCTCAGGCTTCAGAAGAGGCTCGCCTCTAGTGTCCTCCGCTGTGGCAAGAAGAAGGTCTGGTTAGACCCCAATGAGACCAATGAAATCGCCAATGCCAACTCCCGTCAGCAGATCCGGAAGCTCATCAAAGATGGGCTGATCATCCGCAAGCCTGTGACGGTCCATTCCCGGGCTCGATGCCGGAAAAACACCTTGGCCCGCCGGAAGGGCAGGCACATGGGCATAGGTAAGCGGAAGGGTACAGCCAATGCCCGAATGCCAGAGAAGGTCACATGGATGAGGAGAATGAGGATTTTGCGCCGGCTGCTCAGAAGATACCGTGAATCTAAGAAGATCGATCGCCACATGTATCACAGCCTGTACCTGAAGGTGAAGGGGAATGTGTTCAAAAACAAGCGGATTCTCATGGAACACATCCACAAGCTGAAGGCAGACAAGGCCCGCAAGAAGCTCCTGGCTGACCAGGCTGAGGCCCGCAGGTCTAAGACCAAGGAAGCACGCAAGCGCCGTGAAGAGCGCCTCCAGGCCAAGAAGGAGGAGATCATCAAGACTTTATCCAAGGAGGAAGAGACCAAGAAATAAaacctcccactttgtctgtacatactggcctctgtgattacatagatc

agccattaaaataaaacaagccttaatctgccttcc

**>RPL19.dAug10 mRNA Sequence 1068 bp, derived from the genome**
>mRNA RPL19.dAug10, 1068 bp with coding in upper case and exons in alternate colors

gccatgaggtgagggcgagctggtctccatcaggcgctgacgcgtgtcgacaagggactgtcggtcttgggaccgcagctggggttgggggagatgaaatggaggccgccctaaagcggccggtcccggggtttggggtaggccggagcactttcgtcccgggcctccggagtgagggggggcggggagcgtcgcagcaactgagaccaggaaaagtctgccccggctggtgccgcaccgcacacgtgtccggtcgacccacgcgagcagagcaaacggagcgaacaagaccaagccgtgggccctttcttgcttggcacacccggagcggagccgatctctgctttcacgtgatgtagggcaagcctagtgtaggccccaggcctccgactgccgagagagtATGCTCAGGCTTCAGAAGAGGCTCGCCTCTAGTGTCCTCCGCTGTGGCAAGAAGAAGGTCTGGTTAGACCCCAATGAGACCAATGAAATCGCCAATGCCAACTCCCGTCAGCAGATCCGGAAGCTCATCAAAGATGGGCTGATCATCCGCAAGCCTGTGACGGTCCATTCCCGGGCTCGATGCCGGAAAAACACCTTGGCCCGCCGGAAGGGCAGGCACATGGGCATAGGTAAGCGGAAGGGTACAGCCAATGCCCGAATGCCAGAGAAGGTCACATGGATGAGGAGAATGAGGATT

TTGCGCCGGCTGCTCAGAAGATACCGTGAATCTAAGAAGATCGATCGCCACATGTATCACAGCCTGTACCTGAAGGTGAAGGGGAATGTGTTCAAAAACAAGCGGATTCTCATGGAACACATCCACAAGCTGAAGGCAGACAAGGCCCGCAAGAAGCTCCTGGCTGACCAGGCTGAGGCCCGCAGGTCTAAGACCAAGGAAGCACGCAAGCGCCGTGAAGAGCGCCTCCAGGCCAAGAAGGAGGAGATCATCAAGACTTTATCCAAGGAGGAAGAGACCAAGAAATAAaacctcccactttgtctgtacatactggcctctgtgattacatagatcagccattaaaataaaacaagccttaatctgcc

**>RPL19.eAug10 mRNA Sequence 693 bp, derived from the genome**
>mRNA RPL19.eAug10, 693 bp with coding in upper case and exons in alternate colors

agctctttcctttcgctgctgcggccgcagccATGAGGCTTCAGAAGAGGCTCGCCTCTAGTGTCCTCCGCTGTGGCAAGAAGAAGGTCTGGTTAGACCCCAATGAGACCAATGAAATCGCCAATGCCAACTCCCGTCAGCAGATCCGGAAGCTCATCAAAGATGGGCTGATCATCCGCAAGCCTGTGACGGTCCATTCCCGGGCTCGATGCCGGAAAAACACCTTGGCCCGCCGGAAGGGCAGGCACATGGGCATAGGTAAGCGGAAGGGTACAGCCAATGCCCGAATGCCAGAGAAGGTCACATGGATGAGGAGAATGAGGATTTTGCGCCGGCTGCTCAGAAGATACCGTGAATCTAAGAAGATCGATCGCCACATGTATCACAGCCTGTACCTGAAGGTGAAGGGGAATGTGTTCAAAAACAAGCGGATTCTCATGGAACACATCCACAAGCTGAAGGCAGACAAGGCCCGCAAGAAGCTCCTGGTGACCAGGCTGAGGCCCGCAGGTCTAAGACCAAGGAAGCACGCAAGCGCCGTGAAGAGCGCCTCCAGGCCAAGAAGGAGGAGATCATCAAGACTTTATCCAAGGAGGAAGAGACCAAGAAATAAaacctcccactttgtctgtacatactggcctctgtgattacatagatcagccattaaataaaacaagccttaatctgc

**>RPL19.fAug10 mRNA Sequence 383 bp, derived from the genome**
>mRNA RPL19.fAug10, 383 bp with coding in upper case and exons in alternate colors

cgAACATGAGTATGCTCAGGCTTCAGAAGAGGCTCGCCTCTAGTGTCCTCCGCTGTGGCAAGAAGAAGGTCTGGTTAGACCCCAATGAGACCAATGAAATCGCCAATGCCAACTCCCGTCAGCAGATCCGGAAGCTCATCAAAGATGGGCTGATCATCCGCAAGCCTGTGACGGTCCATTCCCGGGCTCGATGCCGGAAAAACACCTTGGCCCGCCGGAAGGGCAGGCACATGGGCATAGGTAAGCGGAAGGGTACAGCCAATGCCCGAATGCCAGAGAAGGTCACATGGATGAGGAGAATGAGGATTTTGCGCCGGCTGCTCAGAAGATACCGTGAATCTAAGAAGATCGATCGCCACATGTAAgcacaccctcttgggccc

**>RPL19.gAug10 mRNA Sequence 1375 bp, derived from the genome**
>mRNA RPL19.gAug10, 1375 bp with coding in upper case and exons in alternate colors

ggaggatcatgagcccagaaattcaagaccggcctgggcaacatagggagactgtctcaaagacaaagggtatttctcacaacctctgtttgtgaaggaaaaagaaaagatagaggttttttgggtttttgaaggcaagaaataagttccagtgtttccatcctttttgagaccgtggggccaagaatgtgagcagtgtctctggcctggcctatttggactctgtgatgtgcttgggccccagttgactgaccaggtgcattatgctttcccaggtcagcagatccggaagctcatcaaagatgggctgatcatccgcaagcctgtgacggtccattcccgggctcgatgccggaaaaacaccttggcccgccggaagggcaggcacatgggcataggtaagtgtggtcatcttctccttaagaaatgataggtgctggcatctatgctgaaatatattcaggatctaagcactctgtctcatcttgagcctgtttcttactccttgatatttgatgtgttttctgcctgtgtgcaaatcagaaagttggtgctggtattggaattgagagtatccctggtaggaggtcacatttactaagtccctacctacactatgccaaggattctgtactttctagtttcagaatgatcgaaggaagctccttacaacgtgctgtgttgccttggtgcactgcct

gttacacccacattctgcatgaagtgaatggggaattattgctgtatttgtgcttcctttaaaattgagtaaacgaaagaacttacgtagttgggatcatcatctggaaaatggaagtaatgctacttaaaatgaggttgtgaggattaaatgagttaaggaccctgacttgaaactatattcacttggtgtacttatacacagtgggcctgggtagtggcccgttcctaactcatcttctccacaggtaagcggaagggtacagccaatgcccgaATGCCAGAGAAGGTCACATGGATGAGGAGAATGAGGATTTTGCGCCGGCTGCTCAGAAGATACCGTGAATCTAAGAAGATCGATCGCCACATGTATCACAGCCTGTACCTGAAGGTGAAGGGGAATGTGTTCAAAAACAAGCGGATTCTCATGGAACACATCCACAAGCTGAAGGCAGACAAGGCCCGCAAGAAGCTCCTGGCTGACCAGGCTGAGGCCCGCAGGTCTAAGACCAAGGAAGCACGCAAGCGCCGTGAAGAGCGCCTCCAGGCCAAGAAGGAGGAGATCATCAAGACTTTATCCAAGGAGGAAGAGACCAAGAAATAAaacctcccactttgtctgtacatactggcctctgtgattacatagatcagccattaaaataaaacaagcctt

**>RPL19.hAug10 mRNA Sequence 577 bp, derived from the genome**
>mRNA RPL19.hAug10, 577 bp with coding in upper case and exons in alternate colors

CtttcctttcgctgctgcggccgcagccATGAGTATGCTCAGGCTTCAGAAGAGGCTCGCCTCTAGTGTCCTCCGCTGTGGCAAGAAGAAGGTCTGGTTAGACCCCAATGAGACCAATGAAATCGCCAATGCCAACTCCCGTCAGCAGATCCGGAAGCTCATCAAAGATGGGCTGATCATCCGCAAGCCTGTGACGGTCCATTCCCGGGCTCGATGCCGGAAAAACACCTTGGCCCGCCGGAAGGGCAGGCACATGGGCATAGGTAAGTGTGGTCATCTTCTCCTTAAGAAATGAtaggtgctggcatctatgctgaaatatattcaggatctaagcactctgtctcatcttgagcctgtttcttactccttgatatttgatgtgttttctgcctgtgtgcaaatcagaaagttggtgctggtattggaattgagagtatccctggtaggaggtcacatttactaagtccctacctacactatgccaaggattctgtactttctagtttcagaatgatcgaaggaagctccttacaacgtgctgtgttgccttggtgcactgcctgttacacccaca

**>RPL19.iAug10-unspliced mRNA Sequence 2362 bp, derived from the genome**
>mRNA RPL19.iAug10-unspliced, 2362 bp with coding in upper case and exons in alternate colors

attctttaaacgctgagtaccagaaatggcataacccccctattcaatccaataagtccttggcttgactttccagaggagaaatgcgaacatgaggctccgagaggtgaaggcatagcgtgggttttgaagtcttaaacccaagggggccagctgcatagcccagagccttaaagatgatttagggaagagtcttatttcgcggctgtggtgtgggtcacaaagggcaggtcttgATGGGGACGTTCATTCTTGCCCAGGATTGGCTTTCAGAGTCTAATCATGTTTTCTGTGTGTCTAGTATGCTCAGGCTTCAGAAGAGGCTCGCCTCTAGTGTCCTCCGCTGTGGCAAGAAGAAGGTCTGGTTAGACCCCAATGAGACCAATGAAATCGCCAATGCCAACTCCCGTGAGTACCTGGGATCTGTCTCTTCACCCTACTTCCTTCTTTTCTCCTGCGTCAGATTAATGATAACACAATTGTGTTGActtttttttttttttttagacagtcttgctctgttgtccaggctggagtgtagtggtgccatctgatcattgcaacccctgctcccaggctcaagtgattctcccacctcagcctcctgagtagctgggattagaggtgtgcaccaccacacccagctaatttactctttatattttattttattttttttgggacagagtctcactttgtc

acccagactggagtgcagtggtaggatctcagctcaccgcaacctctgcctcccaggttcaagcgattctccttcctcagcctcccaagtagctgggactaaggcacgtgccaccacacccagctaatttttgtatctttagtagagatggggtttcatcatgttggtcaggctggtctcaaactcctgacctcaggtcatccatccacctcagcctcccagagtgctgggattatcgacgtgagccaccacacctagcctgggttgacttgttacaaacctaggaaagttcattcagagtgtctataaaatggaggagtcattgtnnnnnnnnnnnnnnnnnnnnnnnnnnnnnnnnnnnnnnnnnnnnnnnnnnnnnnnnnnnnnnnnnnnnnnnnnnnnnnnnnnnnnnnnnnnnnnnnnnnnnnnnnnnnnnnnnnnnnnnnnnnnnnnnnnttttttggagacagagtctcgctctgtcacccaggatggaatgcagtggcacgatcttggttcactgcaatctgctttcgaggttcaagcacttcacctgcctcaccgtctcaagtacctgggattacaggcatgtactaccacgcctggctaatttttgtatttttagtagagatgtggtttcaccgttggccaggctggtctcgaactcctggcctcaagtgatccgcccgcctcagcctctcaaagtgctcagattacaggcctgagccaccacgcccagccatgggtagggagcctttctaagaaagtttttctattctggtctttctttttttccctgtatgtcatcaggatagcccaacagtttatttgccaggcaagttctatctaggaactgcacacaaaaaagtcatggtttaggaagtaattcatgcagcaataggagaaaagaaaggaagctacaagcttagtgaccttgggtggaggaacagctcacaaggcagcctctgatccatcaccaaccagcatctcttcactccgtgtaccctgcaggtatcacagcctgtacctgaaggtgaaggggaatgtgttcaaaaacaagcggattctcatggaacacatccacaagctgaaggcagacaaggcccgcaagaagctcctggcgtaagtttcttttcagagtcttaggggaacattcttagacctttgagagttgttcttagatacttaaaatgtgccaatgcctaagtcttgacttgcacgtagtctgtcttaggaatacagctgttcccttagaggcacccactcctctgtcctgtacacacccactcttcctgtctcgtagggactagaggttagttgaagcaggagcctttggtgggcccagcaactcattccagaagctgactaggctcaaagggagaggtctggggatgtgcttctgtctccctagcatctcatctcttcccaaactgacccgtcttttctcttccctgaccagtgaccaggctgaggcccgcaggtctaagaccaaggaagcacgcaagcgccgtgaagagcgcctccaggccaagaaggaggagatcatcaagactttatccaaggaggaagagaccaagaaataaaacctcccactttgtctgtacatactggcctctgtgattacatagatcagccattaaaataaaacaagccttaatctgc

**Rpl19 mRNA SEQUENCE (Mus musculus)**

According to NCBI gene page:

[**https://www.ncbi.nlm.nih.gov/gene/19921**](https://www.ncbi.nlm.nih.gov/gene/19921)

RPL19 has 2 reference Sequences for mRNA and Protein(s):

[**NM_001159483.1**](https://www.ncbi.nlm.nih.gov/nuccore/NM_001159483.1)**→**[**NP_001152955.1**](https://www.ncbi.nlm.nih.gov/protein/NP_001152955.1)**60S ribosomal protein L19 isoform 2** [**NM_009078.2**](https://www.ncbi.nlm.nih.gov/nuccore/NM_009078.2)**→**[**NP_033104.2**](https://www.ncbi.nlm.nih.gov/protein/NP_033104.2)**60S ribosomal protein L19 isoform 1**

**Using EMBOSS Needle Tool:**

[**https://www.ebi.ac.uk/Tools/services/web/toolresult.ebi?jobId=emboss_needle-I20221004-111505-0196-42953878-p2m**](https://www.ebi.ac.uk/Tools/services/web/toolresult.ebi?jobId=emboss_needle-I20221004-111505-0196-42953878-p2m)

**Pairwise sequence alignement has been performed and common sequence for both isoforms is a s follows:**

########################################

# Program: needle

# Rundate: Tue 4 Oct 2022 11:07:02

# Commandline: needle

# -auto

# -stdout

# -asequence emboss_needle-I20221004-111505-0196-42953878-p2m.asequence

# -bsequence emboss_needle-I20221004-111505-0196-42953878-p2m.bsequence

# -datafile EDNAFULL

# -gapopen 10.0

# -gapextend 0.5

# -endopen 10.0

# -endextend 0.5

# -aformat3 pair

# -snucleotide1

# -snucleotide2

# Align_format: pair

# Report_file: stdout

########################################

#=======================================

#

# Aligned_sequences: 2

# 1: NM_001159483.1

# 2: NM_009078.2

# Matrix: EDNAFULL

# Gap_penalty: 10.0

# Extend_penalty: 0.5

#

# Length: 778

# Identity: 720/778 (92.5%)

# Similarity: 720/778 (92.5%)

# Gaps: 48/778 ( 6.2%)

# Score: 3506.5

#

#

#=======================================

NM_001159483. 1 -----------------ACTGCG-----CATG---------TGCGCCGCG 19

.||||| ||.| |.|||.||.

NM_009078.2 1 GCAGATAATGGGCGGAGCCTGCGGCCCACAAGCTCTTTCCTTTCGCTGCT 50

NM_001159483. 20 GAGTCGGACTGAACCATTTTGCCCGACGAAAGGGTATGCTCAGGCTACAG 69

| |||.|..|.|||| |.|||||||||||||||||

NM_009078.2 51 G---CGGCCGCAGCCAT--------------GAGTATGCTCAGGCTACAG 83

NM_001159483. 70 AAGAGGCTTGCCTCTAGTGTCCTCCGCTGCGGGAAAAAGAAGGTCTGGTT 119

||||||||||||||||||||||||||||||||||||||||||||||||||

NM_009078.2 84 AAGAGGCTTGCCTCTAGTGTCCTCCGCTGCGGGAAAAAGAAGGTCTGGTT 133

NM_001159483. 120 GGATCCCAATGAGACCAATGAAATCGCCAATGCCAACTCCCGTCAGCAGA 169

||||||||||||||||||||||||||||||||||||||||||||||||||

NM_009078.2 134 GGATCCCAATGAGACCAATGAAATCGCCAATGCCAACTCCCGTCAGCAGA 183

NM_001159483. 170 TCAGGAAGCTGATCAAGGATGGGCTGATCATCCGCAAGCCTGTGACTGTC 219

||||||||||||||||||||||||||||||||||||||||||||||||||

NM_009078.2 184 TCAGGAAGCTGATCAAGGATGGGCTGATCATCCGCAAGCCTGTGACTGTC 233

NM_001159483. 220 CATTCCCGGGCTCGTTGCCGGAAAAACACCCTGGCCCGACGGAAGGGCAG 269

||||||||||||||||||||||||||||||||||||||||||||||||||

NM_009078.2 234 CATTCCCGGGCTCGTTGCCGGAAAAACACCCTGGCCCGACGGAAGGGCAG 283

NM_001159483. 270 GCATATGGGCATAGGGAAGAGGAAGGGTACTGCCAATGCTCGGATGCCTG 319

||||||||||||||||||||||||||||||||||||||||||||||||||

NM_009078.2 284 GCATATGGGCATAGGGAAGAGGAAGGGTACTGCCAATGCTCGGATGCCTG 333

NM_001159483. 320 AGAAGGTGACCTGGATGAGAAGGATGAGGATCCTGCGCCGGCTTCTCAGG 369

||||||||||||||||||||||||||||||||||||||||||||||||||

NM_009078.2 334 AGAAGGTGACCTGGATGAGAAGGATGAGGATCCTGCGCCGGCTTCTCAGG 383

NM_001159483. 370 AGATACCGGGAATCCAAGAAGATTGACCGCCATATGTATCACAGCCTGTA 419

||||||||||||||||||||||||||||||||||||||||||||||||||

NM_009078.2 384 AGATACCGGGAATCCAAGAAGATTGACCGCCATATGTATCACAGCCTGTA 433

NM_001159483. 420 CCTGAAGGTCAAAGGGAATGTGTTCAAAAACAAGCGCATCCTCATGGAGC 469

||||||||||||||||||||||||||||||||||||||||||||||||||

NM_009078.2 434 CCTGAAGGTCAAAGGGAATGTGTTCAAAAACAAGCGCATCCTCATGGAGC 483

NM_001159483. 470 ACATCCACAAGCTGAAGGCAGACAAGGCCCGCAAGAAGCTCCTGGCTGAC 519

||||||||||||||||||||||||||||||||||||||||||||||||||

NM_009078.2 484 ACATCCACAAGCTGAAGGCAGACAAGGCCCGCAAGAAGCTCCTGGCTGAC 533

NM_001159483. 520 CAGGCTGAGGCTCGCAGGTCTAAGACCAAGGAAGCACGAAAGCGCCGGGA 569

||||||||||||||||||||||||||||||||||||||||||||||||||

NM_009078.2 534 CAGGCTGAGGCTCGCAGGTCTAAGACCAAGGAAGCACGAAAGCGCCGGGA 583

NM_001159483. 570 GGAGCGCCTCCAGGCCAAGAAGGAAGAGATCATCAAGACTCTGTCCAAGG 619

||||||||||||||||||||||||||||||||||||||||||||||||||

NM_009078.2 584 GGAGCGCCTCCAGGCCAAGAAGGAAGAGATCATCAAGACTCTGTCCAAGG 633

NM_001159483. 620 AGGAGGAGACCAAGAAATAAAGCTTCCCTCGTGTCTGTACATAGCGGCCT 669

||||||||||||||||||||||||||||||||||||||||||||||||||

NM_009078.2 634 AGGAGGAGACCAAGAAATAAAGCTTCCCTCGTGTCTGTACATAGCGGCCT 683

NM_001159483. 670 GGCTGTGGCCTCATGTGGATCAGTCTTTAAAATAAAACAAGCCTTTGTCT 719

||||||||||||||||||||||||||||||||||||||||||||||||||

NM_009078.2 684 GGCTGTGGCCTCATGTGGATCAGTCTTTAAAATAAAACAAGCCTTTGTCT 733

NM_001159483. 720 GTTGCCCTCTTGTTTAGCAAAAAAAAAA 747

||||||||||||||||||||||||||||

NM_009078.2 734 GTTGCCCTCTTGTTTAGCAAAAAAAAAA 761

**Mus musculus ribosomal protein L19 (Rpl19), transcript variant 1, mRNA**

NCBI Reference Sequence: NM_009078.2

GCAGATAATGGGCGGAGCCTGCGGCCCACAAGCTCTTTCCTTTCGCTGCTGCGGCCGCAGCC**ATGAGTATGCTCAGGCTACAGAAGAGGCTTGCCTCTAGTGTCCTCCGCTGCGGGAAAAAGAAGGTCTGGTTGGATCCCAATGAGACCAATGAAATCGCCAATGCCAACTCCCGTCAGCAGATCAGGAAGCTGATCAAGGATGGGCTGATCATCCGCAAGCCTGTGACTGTCCATTCCCGGGCTCGTTGCCGGAAAAACACCCTGGCCCGACGGAAGGGCAGGCATATGGGCATAGGGAAGAGGAAGGGTACTGCCAATGCTCGGATGCCTGAGAAGGTGACCTGGATGAGAAGGATGAGGATCCTGCGCCGGCTTCTCAGGAGATACCGGGAATCCAAGAAGATTGACCGCCATATGTATCACAGCCTGTACCTGAAGGTCAAAGGGAATGTGTTCAAAAACAAGCGCATCCTCATGGAGCACATCCACAAGCTGAAGGCAGACAAGGCCCGCAAGAAGCTCCTGGCTGACCAGGCTGAGGCTCGCAGGTCTAAGACCAAGGAAGCACGAAAGCGCCGGGAGGAGCGCCTCCAGGCCAAGAAGGAAGAGATCATCAAGACTCTGTCCAAGGAGGAGGAGACCAAGAAATAA**AGCTTCCCTCGTGTCTGTACATAGCGGCCTGGCTGTGGCCTCATGTGGATCAGTCTTTAAAATAAAACAAGCCTTTGTCTGTTGCCCTCTTGTTTAGCAAAAAAAAAA

GGTGACCTGGATGAGAAGGA

TTCAGCTTGTGGATGTGCTC

Amplicon size: 162 nts. Intron spanning. Underlined sequence common to both isoforms.

**Further analysis by AceView to check for alternative transcript variants has revealed that Rpl9 (**[**https://www.ncbi.nlm.nih.gov/IEB/Research/Acembly/av.cgi?db=mouse&c=Gene&l=Rpl19**](https://www.ncbi.nlm.nih.gov/IEB/Research/Acembly/av.cgi?db=mouse&c=Gene&l=Rpl19)**). The gene contains**[**8 distinct gt-ag introns**](about:blank)**. Transcription produces**[**5 different mRNAs**](about:blank)**, 4 alternatively spliced variants and 1 unspliced form. Forward and/or Reverse primers are repeated in 3 out of 5 splicing forms corresponding to the longest coding regions sequences.**

**>Rpl19.aSep07 mRNA Sequence 754 bp, derived from the genome**
>mRNA Rpl19.aSep07, 754 bp with coding in upper case and exons in alternate colors

tcgcagataatgggcggagcctgcggcccacaagctctttcctttcgctgctgcggccgcagccATGAGTATGCTCAGGCTACAGAAGAGGCTTGCCTCTAGTGTCCTCCGCTGCGGGAAAAAGAAGGTCTGGTTGGATCCCAATGAGACCAATGAAATCGCCAATGCCAACTCCCGTCAGCAGATCAGGAAGCTGATCAAGGATGGGCTGATCATCCGCAAGCCTGTGACTGTCCATTCCCGGGCTCGTTGCCGGAAAAACACCCTGGCCCGACGGAAGGGCAGGCATATGGGCATAGGGAAGAGGAAGGGTACTGCCAATGCTCGGATGCCTGAGAAGGTGACCTGGATGAGAAGGATGAGGATCCTGCGCCGGCTTCTCAGGAGATACCGGGAATCCAAGAAGATTGACCGCCATATGTATCACAGCCTGTACCTGAAGGTCAAAGGGAATGTGTTCAAAAACAAGCGCATCCTCATGGAGCACATCCACAAGCTGAAGGCAGACAAGGCCCGCAAGAAGCTCCTGGCTGACCAGGCTGAGGCTCGCAGGTCTAAGACCAAGGAAGCACGAAAGCGCCGGGAGGAGCGCCTCCAGGCCAAGAAGGAAGAGATCATCAAGACTCTGTCCAAGGAGGAGGAGACCAAGAAATAAagcttccctcgtgtctgtacatagcggcctggctgtggcctcatgtggatcagtctttaaaataaaacaagcctttgtctgttgccctcttgtttagca

**>Rpl19.bSep07 mRNA Sequence 1424 bp, derived from the genome**
>mRNA Rpl19.bSep07, 1424 bp with coding in upper case and exons in alternate colors

cctccctttctgtcccaaactactctctcagctggtctccctgagcgactctagcttgggccaccggaacttcccggtccctgtgatcctgggacctgcctttaactcctttctccccagtgtcaggcccaggccgccatgaaactagctgagtagacctccctctccaaccgggctctaggtctactggccccagccgggcctggatgatgggctaatctagaaacctttcacagaccgctagccctccgcgaagaccaggccgtccaggccaaaccctcctcacgccgcctgcgcgacttagcgtgacttccggctacccagaggaccacgtgaccaaacgccctcactgcgcatgtgcgccgcggagtcggactgaaccattttgcccgacgaaagggtATGCTCAGGCTACAGAAGAGGCTTGCCTCTAGTGTCCTCCGCTGCGGGAAAAAGAAGGTCTGGTTGGATCCCAATGAGACCAATGAAATCGCCAATGCCAACTCCCGTCAGCAGATCAGGAAGCTGATCAAGGATGGGCTGATCATCCGCAAGCCTGTGACTGTCCATTCCCGGGCTCGTTGCCGGAAAAACACCCTGGCCCGACGGAAGGGCAGGCATATGGGCATAGGGAAGAGGAAGGGTACTGCCAATGCTCGGATGCCTGAGAAGGTGACCTGGATGAGAAGGATGAGGATCCTGCGCCGGCTTCTCAGGAGATACCGGGAATCCAAGAAGATTGACCGCCATATGTATCACAGCCTGTACCTGAAGGTCAAAGGGAATGTGTTCAAAAACAAGCGCATCCTCATGGAGCACATCCACAAGCTGAAGGCAGACAAGGCCCGCAAGAAGCTCCTGGCTGACCAGGCTGAGGCTCGCAGGTCTAAGACCAAGGAAGCACGAAAGCGCCGGGAGGAGCGCCTCCAGGCCAAGAAGGAAGAGATCATCAAGACTCTGTCCAAGGAGGAGGAGACCAAGAAATAAagcttccctcgtgtctgtacatagcggcctggctgtggcctcatgtggatcagtctttaaaataaaacaagcctttgtctgttgccctcttgtttagcagttttggctgctgtgtcttaatgagagctggccacgggaccctgggatgtcagcctagacttcagggtggtattgggacttactgaagggtcacctatttcttgtagccataagtgatgtttctgttctctagagtggctgagtccatcaagcagcctgtgtgctcagatgcttgagaccaacctgccttagcatctcatgaagcattgactgagtggttgttggttatgtagccctggctgtcctggaactcacgctgtagaccaggcttggcctcaaactcagaaatctgcctgcccctgccccccaagtgctgggattaaaggcatgcgccacca

**>Rpl19.cSep07 mRNA Sequence 345 bp, derived from the genome**
>mRNA Rpl19.cSep07, 345 bp with coding in upper case and exons in alternate colors

aagctctttcctttcgctgctgcggccgcagccATGAGTGTCCTCCGCTGCGGGAAAAAGAAGGTCTGGTTGGATCCCAATGAGACCAATGAAATCGCCAATGCCAACTCCCGTCAGCAGATCAGGAAGCTGATCAAGGATGGGCTGATCATCCGCAAGCCTGTGACTGTCCATTCCCGGGCTCGTTGCCGGAAAAACACCCTGGCCCGACGGAAGGGCAGGCATATGGGCATAGGGAAGAGGAAGGGTACTGCCAATGCTCGGATGCCTGAGAAGGTGACCTGGATGAGAAGGATGAGGATCCTGCGCCGGCTTCTCAGGAGATACCGGGAATCCAAGAAGATT

**>Rpl19.dSep07-unspliced mRNA Sequence 1041 bp, derived from the genome**
>mRNA Rpl19.dSep07-unspliced, 1041 bp with coding in upper case and exons in alternate colors

CGGTTCTTGGCCGATGAGGTCGACGGGGCCGACGGGGCCGGCAAGGCTTGGCAGGGATCGGGGCCGGGACCCCGGATGGCAGAGGGGAGAGGGGAGGCGAGCGTGGTGCGGCCGCACCCGGAGCGCGGAGAGCGCGCACGGCCTGGGCCTAGGGAGGGTGGAACGCGGGACGCCGAGGCCCGAAGATCTCCCGCGGGCGCCGCTCTCTGCCTGTCACGCGTTGTGAGGCAGGGCGCGGGGAGAGCAGCGGCGACAGGGCCCTGAgtggctcctcggctcgccggagcccaacagacccctaccatgtaacgtgggacaagtctcgggcaaggccaggcctccgagtgcggaggatgaggtgccgggtcggcttccgcccgaacggctggacgaggtttattaacgtgaaaaagagcccagaaagcaaagagcctcgcctgttctgggtaaacaggctgcgtcacagtcgagtctgatcaattcgtgtctgatccttaacattaaagtcttcaccgagccaagtacccggaagagcttaagcaccccttccccccaccccagatctcaaatggctggccactttggccacgttcattgcccaggtctttcttggtttggatttaaggccttgctttggaaatagttattagaggacaggaaggggtggcaaaaattttaggtctagggggtgtgtttgccc

ttaatagaaatgtatgcatactggtgtatattttagagggaagattttgcttggagtgtttgttgttgttcagatgtcgcttccagggcttactagtgctttctgtgtacccagtatgctcaggctacagaagaggcttgcctctagtgtcctccgctgcgggaaaaagaaggtctggttggatcccaatgagaccaatgaaatcgccaatgccaactcccgtgagtatgatctgtcaccctcactcactatctacctataaggagataagtatgtatgtcttatacatatatcattgtatatagaaatagtttatagggtgcaaccatctcgtgttacat

**>Rpl19.eSep07 mRNA Sequence 363 bp, derived from the genome**
>mRNA Rpl19.eSep07, 363 bp with coding in upper case and exons in alternate colors

aagctctttcctttcgctgctgcggccgcagccATGAGTATGCTCAGGCTACAGAAGAGGCTTGCCTCTAGTGTCCTCCGCTGCGGGAAAAAGAAGGTCTGGTTGGATCCCAATGAGACCAATGAAATCGCCAATGCCAACTCCCGTGAGTATGATCTGTCACCCTCACTCACTATCTACCTATAAggagataagtatgtatgtcttatacatatatcattgtatatagaaatagtttatagggtgcaaccatctcgtgttacattaataatttatgagtaaagtgagtattttatgtcccgtggaagtttttgatatttaaagactggaggatgttggatgaggtttcaagacagaagcaag

1. Sayers EW, Bolton EE, Brister JR, Canese K, Chan J, Comeau DC, Connor R, Funk K, Kelly C, Kim S, Madej T, Marchler-Bauer A, Lanczycki C, Lathrop S, Lu Z, Thibaud-Nissen F, Murphy T, Phan L, Skripchenko Y, Tse T, Wang J, Williams R, Trawick BW, Pruitt KD, Sherry ST. Database resources of the national center for biotechnology information. Nucleic Acids Res. 2022 Jan 7;50(D1):D20-D26. doi: 10.1093/nar/gkab1112. PMID: 34850941; PMCID: PMC8728269.
2. Needleman S.B. and Wunsch C.D. (1970)
   A general method applicable to the search for similarities in the amino acid sequence of two proteins 
   J. Mol. Biol. 48(3):443-53
   PubMed: [5420325](http://europepmc.org/abstract/MED/5420325) 
   DOI: [10.1016/0022-2836(70)90057-4](http://dx.doi.org/10.1016/0022-2836(70)90057-4)
3. Danielle Thierry-Mieg and Jean Thierry-Mieg, [AceView: a comprehensive cDNA-supported gene and transcripts annotation, Genome Biology 2006, 7(Suppl 1):S12](http://genomebiology.com/2006/7/S1/S12)
