# Supplementary material for: Deciphering the sex bias in housekeeping gene expression in adipose tissue: a comprehensive meta-analysis of transcriptomic studies
Source: Biol Sex Differ. 2023 Apr 18;14:20. doi: 10.1186/s13293-023-00506-x (PMC10114345; doi:10.1186/s13293-023-00506-x)
Supplement: Supplementary file 2 — Additional file 2: Table S1. List of primers used for the experimental validation. Table S2. Human sample information. Table S3. Mouse sample information. Table S4. Selection of candidate sex-specific HKGs in gene expression analysis. Fig. S1. Summary of the number of female and male samples found in each Hsa study. Fig. S2. Summary of the number of female and male samples found in each Hsa study. Fig. S3. Variability levels for classic HKGs evaluated in Hsa females. Fig. S4. Variability levels for classic HKGs evaluated in Hsa males. Fig. S5. Variability levels for classic HKGs evaluated in all Mmu samples. Fig. S6. Candidate HKG analysis in mouse adipose tissue, using wt and irs2-/- KO male and female samples. [file 13293_2023_506_MOESM2_ESM.docx]

**Table S1** List of primers used for the experimental validation.

| **Target** | **Species** | **Forward Primer** | **Reverse Primer** |
| --- | --- | --- | --- |
| *18S* | Human | GCAATTATTCCCCATGAACG | GGCCTCACTAAACCATCCAA |
|  | Mouse | AGAAACGGCTACCACATCCA | CATTCCAATTACAGGGCCCG |
| *PPIA* | Human | CCTAAAGCATACGGGTCCTG | TTTCACTTTGCCAAACACCA |
|  | Mouse | AGCATACAGGTCCTGGCATC | TTCACCTTCCCAAAGACCAC |
| *RPL19* | Human | CGAATGCCAGAGAAGGTCAC | CCATGAGAATCCGCTTGTTT |
|  | Mouse | GGTGACCTGGATGAGAAGGA | TTCAGCTTGTGGATGTGCTC |
| *INSR* | Mouse | GGAACGACATTGCCCTGAAG | CCCAGGAGATCTCGGAAGTC |
| *IRS1* | Human | GTTTCCAGAAGCAGCCAGAG | TGAAATGGATGCATCGTACC |
| *LEPR* | Human | CCACCATTGGTACCATTTCC | CCTCATACGAAGACCCAGGA |
|  | Mouse | GGGAATGAGCAAGGTCAAAA | TCAAGTCCCCTTTCATCCAG |
| *PHB* | Mouse | GCATTGGCGAGGACTATGAT | AGCTCTCGCTGGGTAATCAA |
| *PPAR𝛾* | Human | TGCAGTGGGGATGTCTCATA | GGACTCTGGATTCAGCTGGT |

**Table S2** Human sample information. Distribution of the number of samples by study (GSE ID), platform (GPL ID), and sex for Hsa in those studies that included information regarding sex in the GEO entry.

| **GSE ID** | **GPL ID** | **N samples** | **Male** | **Female** |
| --- | --- | --- | --- | --- |
| GSE27657 | GPL570 | 18 | 4 | 14 |
| GSE27916 | GPL570 | 375 | 113 | 262 |
| GSE41168 | GPL570 | 70 | 0 | 70 |
| GSE61302 | GPL570 | 15 | 0 | 15 |
| GSE66159 | GPL570 | 38 | 0 | 38 |
| GSE71416 | GPL570 | 20 | 5 | 15 |
| GSE88837 | GPL570 | 30 | 0 | 30 |
| GSE9624 | GPL570 | 11 | 10 | 1 |
| GSE25401 | GPL6244 | 56 | 0 | 56 |
| GSE25910 | GPL6244 | 36 | 0 | 36 |
| GSE33070 | GPL6244 | 26 | 10 | 16 |
| GSE73655 | GPL6244 | 20 | 6 | 14 |
| GSE41223 | GPL6244 | 20 | 4 | 16 |
| GSE54280 | GPL6244 | 12 | 6 | 6 |
| GSE73108 | GPL10558 | 12 | 0 | 12 |
| GSE65221 | GPL10558 | 136 | 63 | 73 |
| GSE119717 | GPL10558 | 60 | 60 | 0 |
| GSE115645 | GPL10558 | 24 | 21 | 3 |
| GSE43471 | GPL6947 | 96 | 0 | 96 |
| GSE32512 | GPL6947 | 204 | 204 | 0 |
| GSE29231 | GPL6947 | 24 | 0 | 24 |
| GSE29226 | GPL6947 | 24 | 0 | 24 |
| GSE27666 | GPL6947 | 175 | 175 | 0 |
| GSE112307 | GPL6947 | 54 | 0 | 54 |

Distribution of the number of samples by study (GSE ID), platform (GPL ID), and sex for Hsa in those studies that included information regarding sex in the GEO entry.

**Table S3** Mouse sample information. Distribution of the number of samples by study (GSE ID), platform (GPL ID), and sex for Mmu in those studies that included information regarding sex in the GEO entry.

| **GSE ID** | **GPL ID** | **N samples** | **Male** | **Female** |
| --- | --- | --- | --- | --- |
| GSE117352 | GPL1261 | 10 | 10 | 0 |
| GSE140953 | GPL1261 | 12 | 6 | 6 |
| GSE110531 | GPL1261 | 23 | 23 | 0 |
| GSE66132 | GPL1261 | 16 | 16 | 0 |
| GSE77943 | GPL1261 | 21 | 21 | 0 |
| GSE97240 | GPL1261 | 34 | 34 | 0 |
| GSE71367 | GPL1261 | 27 | 27 | 0 |
| GSE67389 | GPL1261 | 16 | 16 | 0 |
| GSE51080 | GPL1261 | 18 | 0 | 18 |
| GSE13432 | GPL1261 | 12 | 12 | 0 |
| GSE38321 | GPL6246 | 10 | 0 | 10 |
| GSE79434 | GPL6246 | 24 | 24 | 0 |
| GSE55272 | GPL6246 | 12 | 12 | 0 |
| GSE37514 | GPL6246 | 15 | 15 | 0 |
| GSE113808 | GPL6885 | 16 | 16 | 0 |
| GSE70857 | GPL6885 | 48 | 48 | 0 |
| GSE57659 | GPL6885 | 179 | 179 | 0 |
| GSE97145 | GPL6887 | 11 | 11 | 0 |
| GSE62612 | GPL6887 | 11 | 11 | 0 |
| GSE50647 | GPL6887 | 42 | 42 | 0 |
| GSE87661 | GPL16570 | 24 | 24 | 0 |
| GSE79711 | GPL16570 | 12 | 12 | 0 |

Distribution of the number of samples by study (GSE ID), platform (GPL ID) and sex for Mmu, *in* those studies that included the information of the sex variable in the GEO entry.

**Table S4** Selection of candidate sex-specific HKGs in gene expression analysis.

| **Gene** | **Relative stability in male** | **Relative stability in females** | **Expression level (TPM)** | **Expression level (TPM) in female** | **Expression level (TPM) in male** |
| --- | --- | --- | --- | --- | --- |
| *ANXA2* | 396,67 | 8.968 | 283,997 | 271,1 | 291,1 |
| *DDX39B* | 68 | 6.633,67 | 197,455 | 192,5 | 201,9 |
| *PLIN4* | 584,33 | 11.594,67 | 499,806 | 441,3 | 530,7 |
| *DNASE2* | 9.911,33 | 349,33 | 38,042 | 37,54 | 38,25 |
| *NDUFB11* | 6.168 | 412,67 | 144,795 | 146,7 | 143,9 |
| *RARA* | 8.267,67 | 306 | 42,575 | 41,94 | 43,71 |

Selection of housekeeping candidate genes proposed to be used as a sex-specific reference in gene expression analysis. These genes are proposed based on their sex-specific values of relative expression stability, obtained from the final MetaRanking positions, and the expression levels have been extracted from GTEx (given in TPM, transcripts per million), which are high enough to be detected by different technologies.


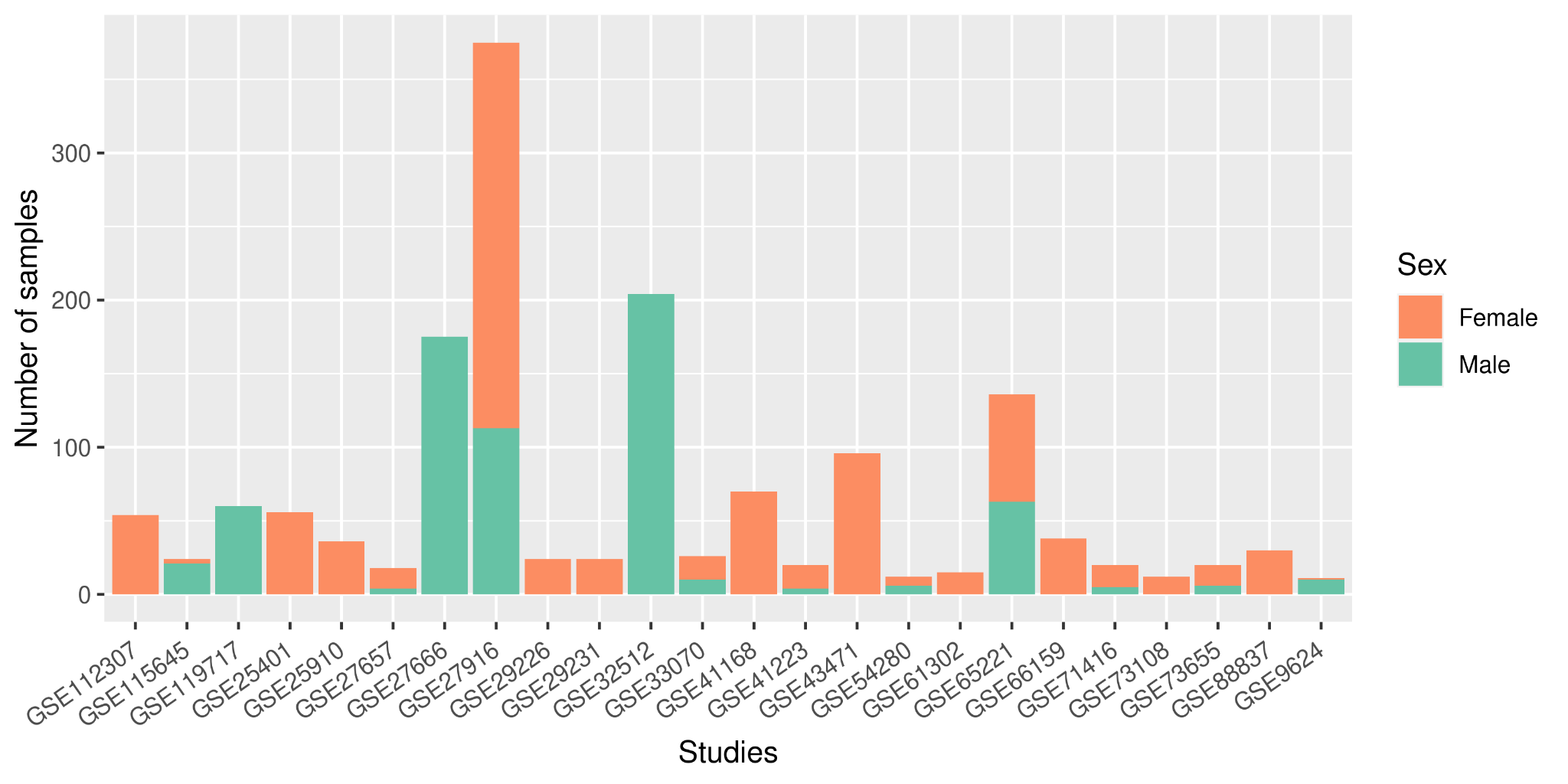


**Fig. S1** Summary of the number of female and male samples found in each Hsa study.


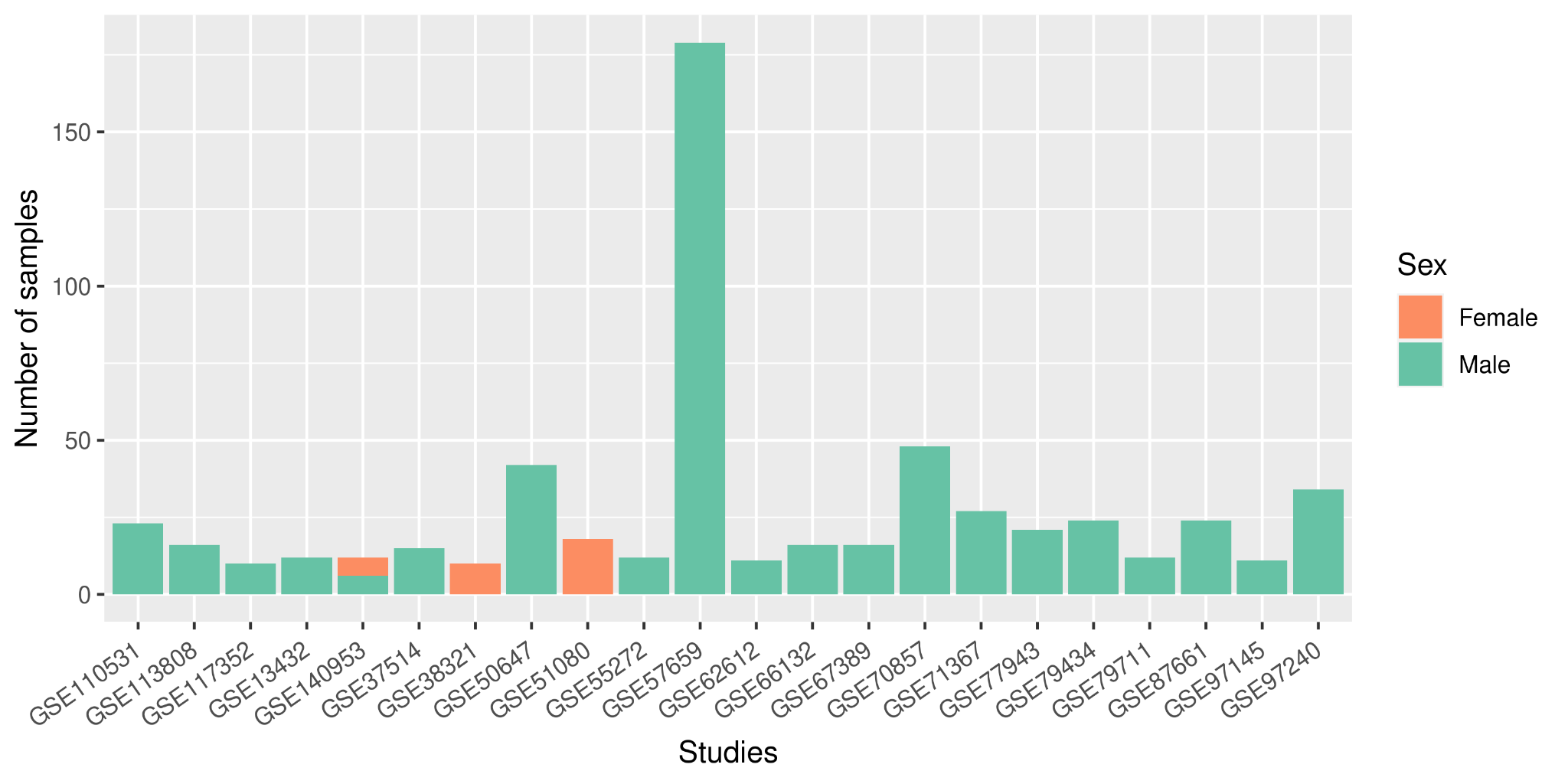


**Fig. S2** Summary of the number of female and male samples found in each Mmu study. One study included samples from both sexes. Most collected samples corresponded to males, evidencing the striking absence of females.


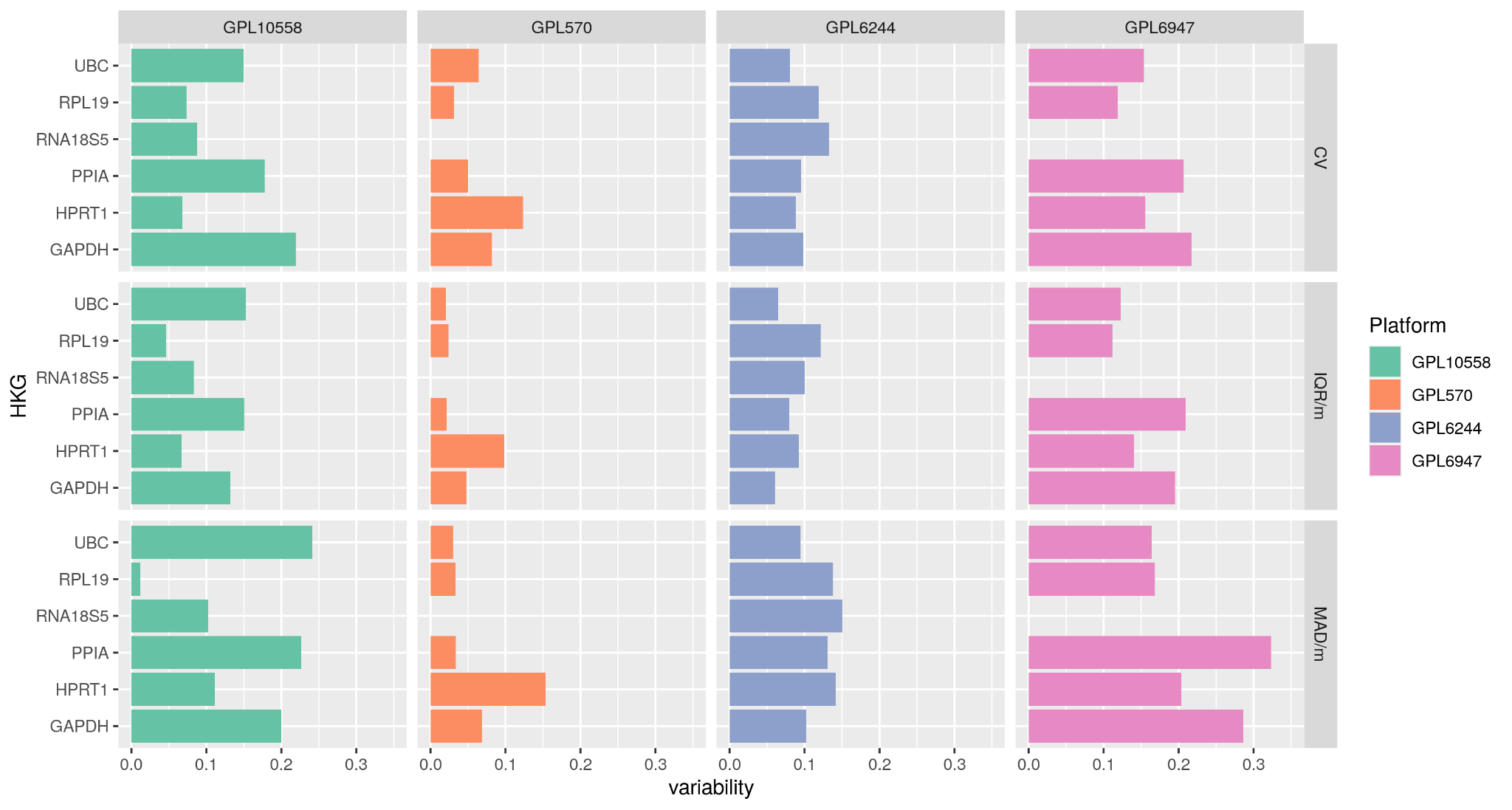


**Fig. S3** Variability levels for classic HKGs evaluated in Hsa females. The variability level found in the selected microarray platforms with the three statistical approaches (CV, IQR/median, and MAD/median) for each HKG is described on the X-axis.


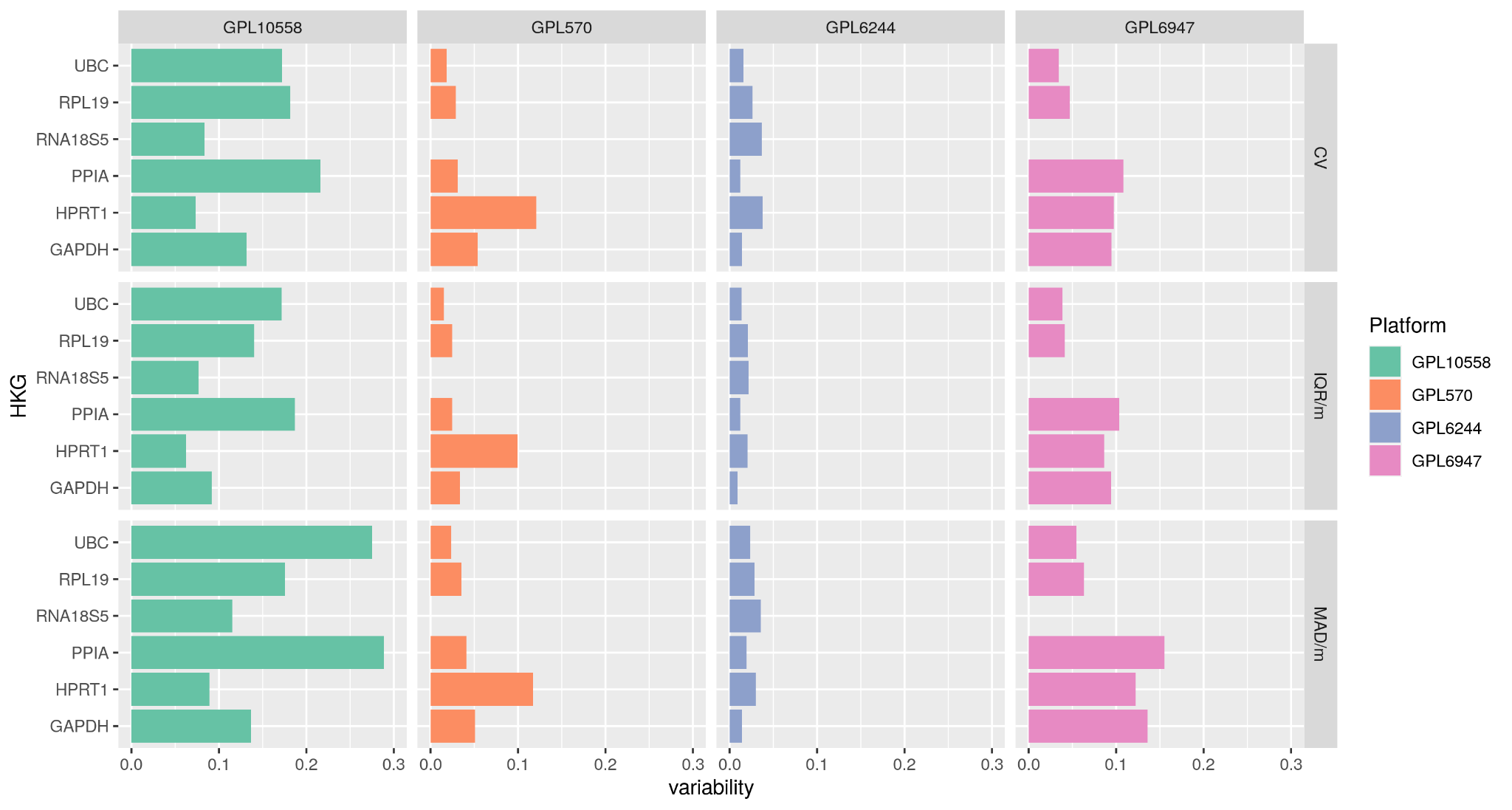


**Fig. S4** Variability levels for classic HKGs evaluated in Hsa males. The variability level found in the selected microarray platforms with the three statistical approaches (CV, IQR/median, and MAD/median) is described on the X-axis for each HKG.


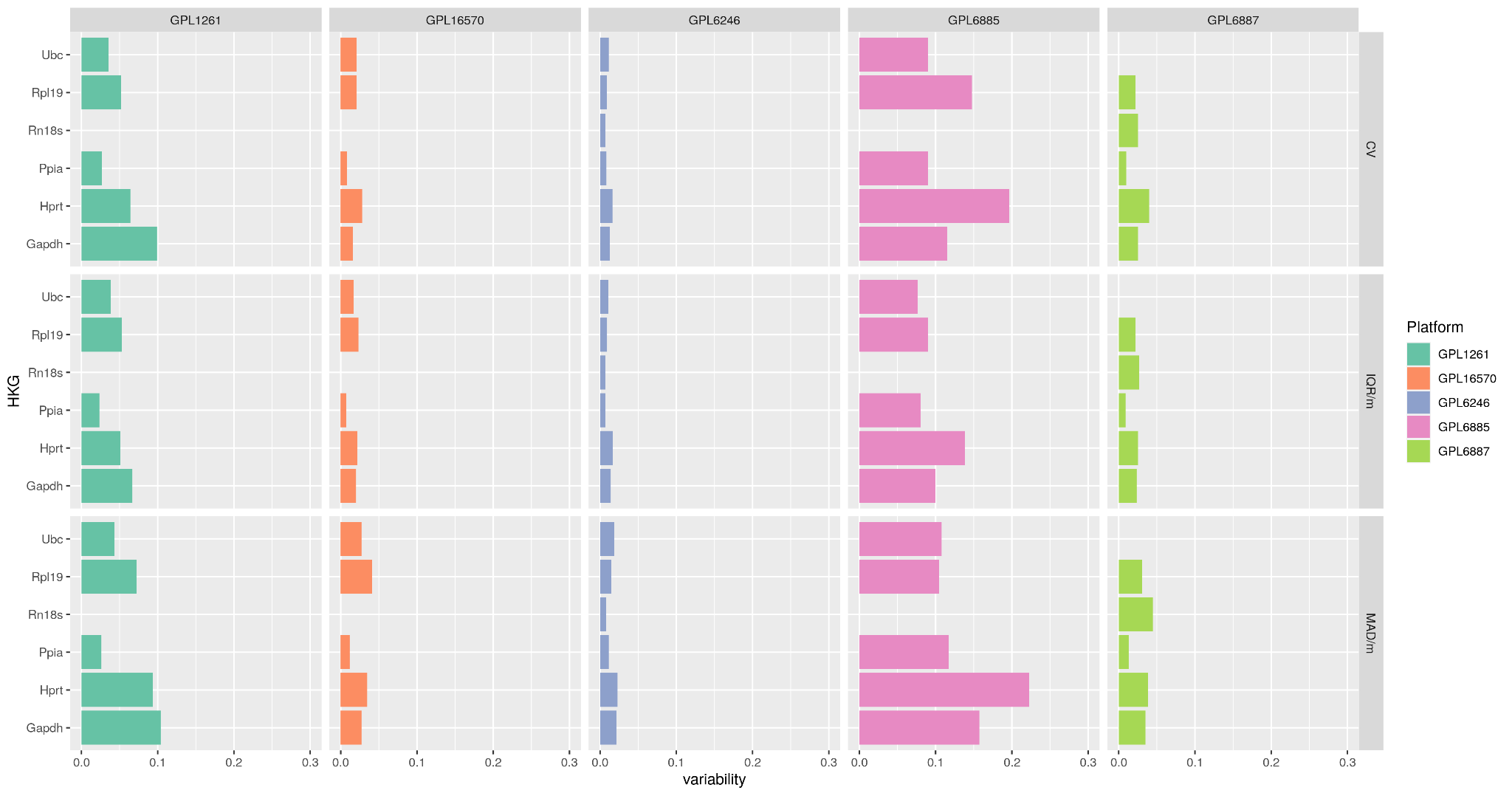


**Fig. S5** Variability levels for classic HKGs evaluated in all Mmu samples. The variability level found in the selected microarray platforms with the three statistical approaches (CV, IQR/median, and MAD/median) is described on the X-axis for each HKG.


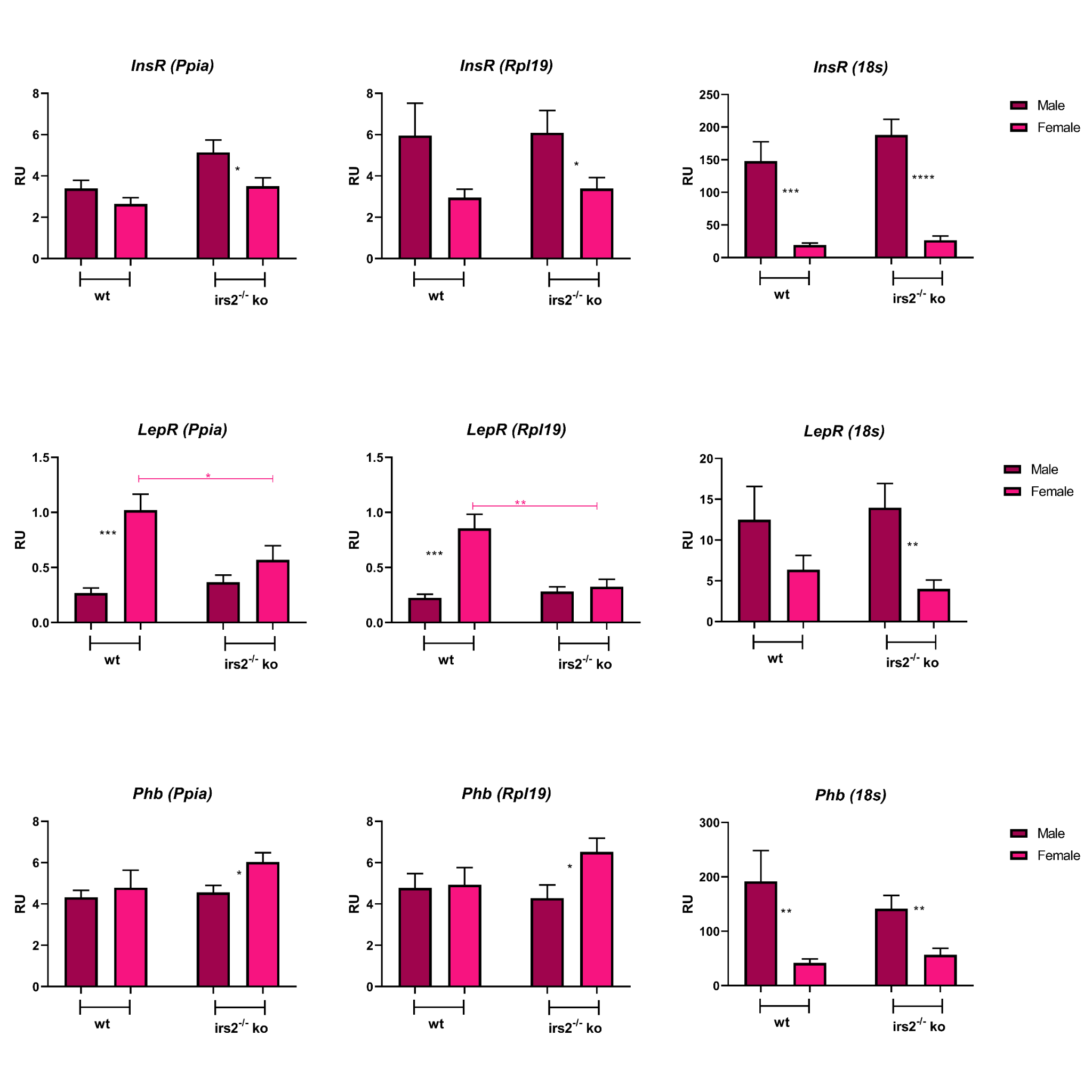


**Fig. S6** Candidate HKG analysis in mouse adipose tissue, using wt and irs2^-/-^ KO male and female samples, showing *Insr*, *Lepr,* and *Phb* gene expression analysis using *Ppia*, *Rpl19,* and *18s* as HKGs. Male wt n=11; Female wt n=13; Male KO n=16 and Female KO n=14. One-way ANOVA and t-test were performed for statistical analysis. The differences observed were considered significant when: p<0.05 (*), p<0.01 (**), and p<0.001 (***).
