# Supplementary material for: Deciphering the sex bias in housekeeping gene expression in adipose tissue: a comprehensive meta-analysis of transcriptomic studies
Source: Biol Sex Differ. 2023 Apr 18;14:20. doi: 10.1186/s13293-023-00506-x (PMC10114345; doi:10.1186/s13293-023-00506-x)
Supplement: Supplementary file 3 — Additional file 3. Methods and results of deconvolution analysis. [file 13293_2023_506_MOESM3_ESM.html]

Deciphering the Sex Bias in Housekeeping Gene Expression in Adipose Tissue: a Comprehensive Meta-analysis of Transcriptomic Studies


# Deciphering the Sex Bias in Housekeeping Gene Expression in Adipose Tissue: a Comprehensive Meta-analysis of Transcriptomic Studies

**Authors:** Maria Guaita-Cespedes1,2,+, Rubén Grillo-Risco1,+, Marta R. Hidalgo1, Sonia Fernández-Veledo3,4, Deborah Jane Burks4,5, María de la Iglesia-Vayá6, Amparo Galán4,5,\*, and Francisco Garcia-Garcia1,\*

1Bioinformatics and Biostatistics Unit, Principe Felipe Research Center (CIPF), Valencia, 46012, Spain.

2Hematology Research Group, Instituto de Investigación Sanitaria La Fe (IIS La Fe), Valencia, 46026, Spain.

3Department of Endocrinology and Nutrition and Research Unit, University Hospital of Tarragona Joan XXIII, Institut d’Investigaciò Sanitària Pere Virgili (IISPV), Tarragona, Spain.

4CIBER de Diabetes y Enfermedades Metabólicas Asociadas (CIBERDEM), Instituto de Salud Carlos III, Madrid, Spain.

5Molecular Neuroendocrinology Laboratory, Principe Felipe Research Center (CIPF), Valencia, 46012, Spain.

6Imaging Unit FISABIO-CIPF, Fundación para el Fomento de la Investigación Sanitaria y Biomédica de la Comunidad Valenciana, 46012, Valencia, Spain.

+These authors contributed equally to this work

**\*Corresponding authors:**

Francisco García-García, Bioinformatics and Biostatistics Unit, Príncipe Felipe Research Center, C/ Eduardo Primo Yúfera, 3. 46012 Valencia, Spain. Tel: (+34) 963289680, Fax: (+34) 963289701. E-mail: fgarcia@cipf.es

Amparo Galán, Molecular Neuroendocrinology Laboratory, Príncipe Felipe Research Center, C/ Eduardo Primo Yúfera, 3. 46012 Valencia, Spain. Tel: (+34) 963289680, Fax: (+34) 963289701. E-mail: agalan@cipf.es

Evaluation of sex differences in cell composition

# 1 Methods

To identify differences in the abundance of cell composition between males and females, we first performed a deconvolution analysis for each dataset that included samples of both sexes. The deconvolution analysis was performed with MCP-count since it allows using a normalized gene expression matrix as input. The scores returned by MCP-count are expressed in arbitrary units, allowing the abundance comparison between samples. MCP-count estimates the relative abundance of diverse immune and stromal populations in heterogeneous bulk samples but also allows the analysis of new cell types, including new transcriptomic markers. The selected genes for the analysis of adipocytes and adipose stem precursors cell types are shown in Supplemental material 2.1 section. Then, we performed a Wilcoxon test to compare the abundance means in cell types between sexes. Finally, we counted how many times there are significant differences in abundance means for each cell type across datasets.

# 2 Results

## 2.1 Selection of Transcriptomic markers

Table showing the selected transcriptomic markers for each cell type and source.

## 2.2 Deconvolution analysis

Deconvolution analysis results for each dataset, showing the cell type, abundance score and sex per sample.

### GPL570\_GSE27657

### GPL570\_GSE27916

### GPL570\_GSE71416

### GPL6244\_GSE33070

### GPL6244\_GSE73655

### GPL6244\_GSE41223

### GPL6244\_GSE54280

### GPL10558\_GSE65221

### GPL10558\_GSE115645

## 2.3 Wilcoxon test

Wilcoxon test results for each dataset, showing the statistic and p-value per cell type (p-values lower than 0.05 are shown in green).

### GPL570\_GSE27657

#### Wilcoxon test

#### Boxplot

### GPL570\_GSE27916

#### Wilcoxon test

#### Boxplot

### GPL570\_GSE71416

#### Wilcoxon test

#### Boxplot

### GPL6244\_GSE33070

#### Wilcoxon test

#### Boxplot

### GPL6244\_GSE73655

#### Wilcoxon test

#### Boxplot

### GPL6244\_GSE41223

#### Wilcoxon test

#### Boxplot

### GPL6244\_GSE54280

#### Wilcoxon test

#### Boxplot

### GPL10558\_GSE65221

#### Wilcoxon test

#### Boxplot

### GPL10558\_GSE115645

#### Wilcoxon test

#### Boxplot

## 2.4 Summary results

Table summarizing the number of times in which a cell type has a differential abundance between sexes across datasets. Overall, we did not find differences between the sexes in cellular composition. Only 2 of 9 datasets indicated a higher abundance of adipocytes in females. We found significant differences in adipocyte stem progenitor cells in 1/3 of the datasets, but with contradictory results. In two cases the mean was higher in females and one case in males.

# 3 References

- Becht, E., Giraldo, N. A., Lacroix, L., Buttard, B., Elarouci, N., Petitprez, F., … & De Reyniès, A. (2016). Estimating the population abundance of tissue-infiltrating immune and stromal cell populations using gene expression. Genome biology, 17(1), 1-20.
- Duerre, D. J., & Galmozzi, A. (2022). Deconstructing Adipose Tissue Heterogeneity One Cell at a Time. Frontiers in Endocrinology, 467.
- Lenz, M., Arts, I. C., Peeters, R. L., de Kok, T. M., & Ertaylan, G. (2020). Adipose tissue in health and disease through the lens of its building blocks. Scientific reports, 10(1), 1-14.
- Bäckdahl, J., Franzén, L., Massier, L., Li, Q., Jalkanen, J., Gao, H., … & Mejhert, N. (2021). Spatial mapping reveals human adipocyte subpopulations with distinct sensitivities to insulin. Cell metabolism, 33(9), 1869-1882.
